# Prokaryotic Response to Phytodetritus-Derived Organic Material in Epi- and Mesopelagic Antarctic Waters

**DOI:** 10.3389/fmicb.2020.01242

**Published:** 2020-06-09

**Authors:** Vincenzo Manna, Francesca Malfatti, Elisa Banchi, Federica Cerino, Fabio De Pascale, Annalisa Franzo, Riccardo Schiavon, Alessandro Vezzi, Paola Del Negro, Mauro Celussi

**Affiliations:** ^1^Oceanography Division, Istituto Nazionale di Oceanografia e di Geofisica Sperimentale – OGS, Trieste, Italy; ^2^Department of Life Sciences, Università degli Studi di Trieste, Trieste, Italy; ^3^Scripps Institution of Oceanography, University of California, San Diego, San Diego, CA, United States; ^4^Department of Biology, Università degli Studi di Padova, Padua, Italy

**Keywords:** particulate organic matter, particle-attached, free-living, 16S rRNA, microbial community, extracellular enzymes, carbon cycle, Southern Ocean

## Abstract

Particulate organic matter (POM) export represents the underlying principle of the biological carbon pump, driving the carbon flux from the sunlit to the dark ocean. The efficiency of this process is tightly linked to the prokaryotic community, as >70% of POM respiration is carried out by particle-associated prokaryotes. In the Ross Sea, one of the most productive areas of the Southern Ocean, up to 50% of the surface primary production is exported to the mesopelagic ocean as POM. Recent evidence suggests that a significant fraction of the POM in this area is composed of intact phytoplankton cells. During austral summer 2017, we set up bottle enrichment experiments in which we amended free-living surface and deep prokaryotic communities with organic matter pools generated from native microplankton, mimicking the particle export that may derive from mild (1 μg of Chlorophyll *a* L^–1^) and intense (10 μg of Chlorophyll *a* L^–1^) phytoplankton bloom. Over a course of 4 days, we followed free-living and particle-attached prokaryotes’ abundance, the degradation rates of polysaccharides, proteins and lipids, heterotrophic production as well as inorganic carbon utilization and prokaryotic community structure dynamics. Our results showed that several rare or undetected taxa in the initial community became dominant during the time course of the incubations and that different phytodetritus-derived organic matter sources induced specific changes in microbial communities, selecting for peculiar degradation and utilization processes spectra. Moreover, the features of the supplied detritus (in terms of microplankton taxa composition) determined different colonization dynamics and organic matter processing modes. Our study provides insights into the mechanisms underlying the prokaryotic utilization of phytodetritus, a significant pool of organic matter in the dark ocean.

## Introduction

Whether organic particles derive from phytoplankton, zooplankton or dissolved organic matter (DOM) aggregation, they represent a hotspot of microbial activity in aquatic ecosystems (Grossart and Simon, 1993; Kiørboe, 2001). To process and consume the particulate organic matter (POM), particle-associated microbes need a complex suite of enzymes that hydrolyze high molecular weight (HMW) substrates, but not all microorganisms possess the enzymatic capabilities to hydrolyze all organic matter moieties (Kiørboe et al., 2002; Arnosti, 2011). On one hand, this means that the POM quality selects for a subset of microbes, capable to degrade that specific pool of organic matter (Kiørboe et al., 2003). On the other hand, selective degradation of particles’ constituents leads to shifts in quality and quantity of carbon, driving changes in the particle-associated community over time (Datta et al., 2016). Therefore, changes in POM composition will affect the dynamics of the associated communities (Grossart, 1999; Eiler and Bertilsson, 2004; Grossart et al., 2005; LeCleir et al., 2014).

The export of POM represents the underlying principle of the biological carbon pump which annually removes about one third of anthropogenic atmospheric CO_2_ via the export of organic carbon produced by phytoplankton in the euphotic ocean toward the deep ocean (Sabine et al., 2004; Boyd et al., 2019). The POM settling rate is a central factor in determining the efficiency of carbon sequestration: the deeper the particles sink, the longer the carbon of which they are made will be removed from the atmospheric and upper-oceanic reservoirs (Kwon et al., 2009; Passow and Carlson, 2012). However, only a small fraction, between 5 and 25% of the POM produced in the upper ocean, reaches the mesopelagic realm (De La Rocha and Passow, 2007; Buesseler and Boyd, 2009). Organic particles are indeed subjected to remineralization as they sink to the ocean interior, leading to the release of CO_2_ and DOM and causing a reduction in the biological carbon pump efficiency (De La Rocha and Passow, 2007). Remineralization processes are mainly carried out by particle-associated prokaryotes, which can respire more than 70% of the sinking POM (Giering et al., 2014).

The Southern Ocean (SO) makes up approximately 10% of the world’s ocean (Rogers et al., 2020), yet it is responsible for the ventilation of the global ocean as well as for a conspicuous drawdown (ca. 10%) of the anthropogenic CO_2_ emissions (Turner et al., 2009; Hauck et al., 2015). The SO is a high nutrient-low chlorophyll system because of the limitation of the primary producer growth by micronutrients such as iron (Strzepek et al., 2011; Boyd et al., 2012). Nevertheless, its coastal, shallower zones represent hotspots of primary production (Smetacek and Nicol, 2005). Among them, the Ross Sea is widely recognized as one of the most productive sectors of the SO, supporting 1/3 of its total annual productivity and accounting for more than 25% of its total CO_2_ uptake (Arrigo et al., 2008; Smith et al., 2014). About half of the carbon fixed into biomass by primary producers in the Ross Sea surface layer is exported as POM in the mesopelagic system, a flux that may represent up to 40% of the global POM export (Ducklow et al., 2001; Catalano et al., 2010). Cumulative evidence demonstrates that a significant fraction of this POM is represented by healthy or at least intact phytoplankton cells (DiTullio et al., 2000; Rembauville et al., 2015; Zoccarato et al., 2016). Furthermore, Agusti et al. (2015) demonstrated that the presence at depth of phytoplankton is widespread at a global scale, unraveling a previously overlooked source of organic matter in the dark ocean.

While marine snow associated community dynamics are well characterized by both experimental and environmental studies (e.g., Bižić-Ionescu et al., 2015, Bižic-Ionescu et al., 2018; Fontanez et al., 2015; Datta et al., 2016; Pelve et al., 2017; Duret et al., 2019), limited information exist on the degradation mechanisms of phytodetrital particles and on their associated prokaryotic communities (Becquevort and Smith, 2001; Bidle et al., 2002). To fill this gap, during austral summer 2017, we performed 8 microcosm incubation experiments by providing freshly produced algal detritus, generated from on-site collected microplankton net tows, to free-living prokaryotic communities (<1 μm) from the surface and from the mesopelagic zone (548 to 1051 m) of a coast-offshore transect in the Ross Sea. We measured the functional (i.e., extracellular enzymatic activities, heterotrophic carbon production and dark dissolved inorganic carbon uptake) as well as the taxonomic community response (16S rRNA Illumina amplicon sequencing) under the hypothesis that different detrital pools yield distinct metabolic and community shifts.

## Materials and Methods

### Sampling

The sampling stations (Table 1 and Supplementary Figure 1) were placed along a transect heading N-E from Terra Nova Bay based on previous studies (e.g., Celussi et al., 2009; Zoccarato et al., 2016). The sampling was performed during the XXXII Italian Antarctic Expedition in 2017. Water samples were collected by means of 12-L Niskin bottles mounted on a Rosette carousel equipped with a SBE 9/11 Plus CTD profiler. Samples were collected at surface (ca. 2 m) and at the bottom layer (ranging from 548 to 1051 m) at each of the four stations. Right after the CTD casts, 7 plankton tows were performed at each station by means of a 20-μm mesh-sized net in order to collect a final plankton sample volume equal to 1.5 L. The depth of the net deployment was chosen according to the CTD chlorophyll *a* fluorescence profiles and ranged between 60 and 120 m (Supplementary Figure 2).

**TABLE 1:** Details of the sampling stations (dates, coordinates, bottom depth, and sampling depth) in the Ross Sea.

Station	Date	Longitude (°E)	Latitude (°N)	Bottom depth	Sampling depths
B	Jan 17, 2017	175.089	–74	578 m	∼2; 565 m
C1	Feb 01, 2017	170.9092	–74.1877	563 m	∼2; 548 m
C2	Jan 23, 2017	166.8105	–74.75716	905 m	∼2; 899 m
D	Jan 08, 2017	164.5333	–75.12672	1057 m	∼2; 1051 m

### Experimental Design and Setup

Microcosm (HCl-washed 2-L Nalgene PC bottles) experiments were set-up on board the R/V Italica. Seawater was filtered through 1 μm PC filters (Whatman) in order to keep free-living prokaryotes only. The pore-size was chosen considering the cell-size frequency distribution study by La Ferla et al. (2015) in the same geographical area. For each experiment, 1 μm-filtered seawater from surface and bottom layers of the four stations was amended with phytodetritus in order to achieve final concentrations of 1−10 μg of Chlorophyll *a* equivalent per liter (hereinafter 1−10 μg L^–1^, respectively). Unamended 1 μm-filtered seawater was used as control. A conceptual scheme of the experimental setup is depicted in Supplementary Figure 3. All treatments and controls were run in experimental duplicates and were incubated in the dark, at *in situ* temperature for 4 days. Sampling within the microcosms were performed at day (d) 0, right after the amendments, and after 1, 2, and 4 days.

The phytodetritus was generated by plankton net samples through 7 cycles of freezing (−80°C)/thawing (80°C) (Bidle and Azam, 2001). Unwashed aliquots of the detritus were then added to the 2-L microcosms containing 1 μm-filtered seawater at 1 and 10 μg L^–1^ of Chlorophyll *a* equivalent (see section “Chemical Analysis”). These pigment concentrations were selected in order to mimic mild and dense bloom conditions (e.g., Schine et al., 2016) in the Ross Sea.

At every time point, samples were collected for the determination of free-living heterotrophic prokaryote abundance, viral abundance, heterotrophic carbon production rates and the activity of the exoenzymes β-glucosidase, lipase, and leucine aminopeptidase. Additional aliquots were collected at d0, and after 1 and 4 days for the estimation of the abundance of particle-attached prokaryotes. Dark Dissolved Inorganic Carbon (DDIC) fixation rates were measured only at d0 since the incubation time for the analysis was 96 h (see section “Microbial Metabolic Activities”). At the end of the experiments, the remaining seawater volume in the PC bottles was >1.75 L. At d0 and at the end of the experiments, DNA was collected as described in Section “DNA Extraction, Amplicon Library Preparation, and Sequencing.”

### Chemical Analysis

Chlorophyll *a* concentration in the net samples was determined by the fluorometric method by Lorenzen and Jeffrey (1980). Briefly, triplicate 3 mL aliquots were filtered onto glass fiber filters (Whatman GF/F) and the extraction was performed with 90% v/v acetone at 4°C in the dark for 4 h (Holm-Hansen et al., 1965). Holm-Hansen et al. (2000) proposed to use at least 2-h extraction incubation prior fluorometric reading for Antarctic seawater samples. This quick procedure was chosen in order to expedite the experimental setup since we needed to titrate the phytodetritus prior amendment. Fluorometric reads were performed before and after acidification with two drops of HCl 1N by means of a Shimadzu RF-1501 spectrofluorometer at 450 nm excitation and 665 nm emission wavelength. Calibration curves were made with pure Chlorophyll *a* standard from spinach (Sigma-Aldrich).

The analysis of particulate and dissolved organic C concentration within the generated phytodetritus was carried out by standard protocols, as detailed in the section “Supplementary Methods.”

### Phytodetritus Composition

The analysis of microplankton net tows was carried out by an inverted microscope (LEICA DMi8) equipped with phase contrast after fixation with neutralized formaldehyde (1.6% final concentration, f.c., Throndsen, 1978). To obtain the relative abundance of microplankton taxa, the samples were allowed to settle in a Utermöhl chamber and examined following the Utermöhl method (Utermöhl, 1958). Cell counts were performed along transects or fields at a magnification of 400× counting a minimum of 200 cells.

### Heterotrophic Prokaryotes and Viruses

The abundance of free-living heterotrophic prokaryotes (FL-HP) and of virus-like particles (VLP) was estimated by flow cytometry. A FACSCanto II (Becton Dickinson) instrument was used, equipped with an air-cooled laser at 488 nm and standard filter setup. Samples (1.7 mL) were fixed with 0.5% (f.c.) glutaraldehyde solution (Grade I for EM analyses, Sigma Aldrich). Fixed samples were kept at 4°C for approximately 15 mins and then stored at −80°C until analysis (Brussaard, 2004). Prior to enumeration, samples were thawed at room temperature and diluted 1:10 (FL-HP) and 1:50 (VLP) with 0.2 μm-filtered Tris-EDTA buffer 1× (Sigma Aldrich). Then samples were stained with SYBR Green I nucleic acid dye (Life Technologies), according to Marie et al. (1999); Brussaard (2004) for FL-HP and VLP, respectively. FL-HP were stained (1×, f.c.) and incubated for 10 mins in the dark at room temperature. Virus-like particles were stained (0.5×, f.c.) and incubated for 15 mins in the dark at 80°C. Total virus abundance was obtained by correcting the total count for noise, with 0.2 μm-filtered Tris-EDTA buffer 1× (Sigma Aldrich) as blank. Data were acquired and processed with the FACSDiva software (Becton Dickinson). The flow rate was calibrated daily, by running distilled water and weighing it before and after the run (at least 5 replicates). Abundances were then calculated using the acquired cell counts and the respective flow rates.

From the same glutaraldehyde-fixed samples, particle-attached (PA) prokaryotes were counted at the home laboratory by epifluorescence microscopy after staining the cells with 4,6-diamidino-2-phenylindole (DAPI, Sigma Aldrich) following the protocol of Porter and Feig (1980) with slight modifications, as reported in Celussi et al. (2017a). Briefly, 1.5 mL aliquots were filtered in duplicates onto 0.2 μm black polycarbonate membranes (Whatman) that were subsequently placed on a drop (50 μL) of DAPI (30 μg mL^–1^ in an autoclaved 3.7% NaCl solution) for 15 min in the dark. The back of the filters were gently dried onto a kimwipe tissue, mounted between layers of immersion oil (Type A, Cargille) and stored at −20°C. Particle-attached prokaryotes (PA) were counted at 1000× magnification (Olympus BX 60 FS) under a UV filter set (BP 330−385 nm, BA 420 nm). A minimum of 300 cells was counted for each membrane in at least 20 randomly selected fields.

### Microbial Metabolic Activities

Extracellular enzymatic activities (EEAs) were tested using fluorogenic substrate analogs (Hoppe, 1993) derived from 7-amino-4-methylcoumarin (AMC) and 4-methylumbelliferone (MUF). Leucine-aminopeptidase activity (AMA) was assayed as the hydrolysis rate of leucine-AMC. β-glucosidase (BGLU), and lipase (LIP) activities were assayed using MUF-β-D-glucoside and MUF-oleate (Sigma Aldrich), respectively. Hydrolysis was measured by incubating 2.5 mL subsamples with 200 μM leucine-AMC, MUF-β-D-glucoside, 100 μM MUF-oleate (saturating final concentrations, Celussi et al., 2009) for 3–7 h in the dark at *in situ* temperature. Fluorescence increase due to AMC and MUF hydrolyzed from the model substrates was measured using a Shimadzu RF-1501 spectrofluorometer (AMC = 380 nm excitation and 440 nm emission; MUF = 365 nm excitation and 455 nm emission). Triplicate calibration curves were performed daily, using 0.2 μm-filtered seawater and 5 μM standard solutions of AMC and MUF (Sigma Aldrich). EEAs were measured also on diluted (1:1000) aliquots of detritus, and hydrolysis rates were not measurable or negligible (<0.01 nM h^–1^).

Heterotrophic carbon production (HCP) was measured with the method of ^3^H-leucine (Leu) incorporation (Kirchman et al., 1985). Triplicate 1.7 mL subsamples and one killed control (5% trichloroacetic acid − TCA − f.c.) were amended with 20 nM radiotracer (50.2 Ci mmol^–1^; Perkin Elmer) and incubated for 3–7 h in the dark at *in situ* temperature. The extraction of ^3^H-labeled proteins was carried out following the microcentrifugation method (Smith et al., 1992). After the addition of 1 mL of scintillation cocktail (Ultima Gold^TM^ MV; Packard), the activity was determined by a TRI-CARB 2900 TR Liquid Scintillation Analyzer.

Rates of Dark Dissolved Inorganic Carbon fixation (DDIC) were determined by the incorporation of NaH^14^CO_3_ (Herndl et al., 2005; Yakimov et al., 2011), as described previously (Celussi et al., 2017b). Samples were collected from the microcosms at d0 in 2 replicates (40 mL each) plus one killed control (treated with 2% formalin, f.c.). Each tube was spiked with 100 μL of a NaH^14^CO_3_ solution (42.1 mCi mmol^–1^; DHI) to yield a final activity of 0.25 μCi mL^–1^. Samples were incubated in the dark for 96 h at *in situ* temperature and then fixed with 2% dolomite-buffered formalin. The whole volume in each tube was filtered through 0.2 μm-pore-size polycarbonate membranes (Whatman). Filters were washed twice with 10 mL of an autoclaved NaCl (3.8% w/v) solution, acidified with HCl fumes in scintillation vials for 12–16 h and frozen at −20°C. Once in the laboratory, the scintillation vials were filled with 5 mL scintillation cocktail (Filter-Count^TM^, Perkin Elmer) and the activity was determined by a TRICARB 2900 TR Liquid Scintillation Analyzer. The filtration and the acidification steps were performed within 24 h from fixation with formalin. DDIC was determined according to the following equation (Steeman Nielsen, 1952):

$$DDIC=\frac{DIC*DPM_{sample-control}*1.05*12}{DPM_{added}*T}$$

where *DIC* is the concentration of dissolved inorganic carbon in samples (2.16 and 2.24 mmol L^–1^, for surface and bottom experiments respectively, Ingrosso, unpublished data from previous surveys in the Ross Sea), 1.05 is the correction factor for slower assimilation of ^14^C than ^12^C, 12 is the molecular weight of C, *DPM*_**sample–control**_ are the DPM measured in every replicate corrected for the ones in the control, *DPM*_**added**_ is the activity (certified by the provider) of the NaH^14^CO_3_ solution spiked in each tube and *T* is the incubation time.

### DNA Extraction, Amplicon Library Preparation, and Sequencing

At the beginning of the experiments the native free-living prokaryotic community (1 μm-filtered) was collected on 0.2 μm polyether-sulfone membrane filters (SUPOR 200, Pall) and stored at −80°C for DNA analyses. The filtered volumes were 2 to 4 L for surface experiments and 8 L for deep water experiments. The same protocol was used at the end of the experiments (d4), by filtering the remaining volume in each bottle, approximately 1.75 L. DNA was extracted using the DNeasy PowerWater kit (Qiagen) following the manufacturer’s instructions. The quantity of DNA extracts was measured by Qubit Fluorometer (Thermo Fisher Scientific). Extracted DNA was stored at −20°C until further analysis.

To generate prokaryotic barcodes, the extracted DNA was PCR-amplified by using the primer pair 515F-Y (5′-GTGYCAGC MGCCGCGGTAA-3′) and 926R (5′-CGYCAATTYMTTTRA GTTT-3′), which encompass the V4 and V5 hypervariable loops of 16S rRNA genes (Parada et al., 2016). PCR mixtures (25 μL final volume) were as follows: 5 ng of template DNA, 0.5 U of Phusion High-Fidelity DNA polymerase (Thermo Fisher Scientific), 1 × Phusion HF buffer, 200 μM of each dNTP, and 0.5 μM of each primer. PCR amplifications (98°C for 4 min; 25 cycles of 98°C for 20 s, 57°C for 30 s, 72°C for 30 s; 72°C for 5 min) were carried out in duplicate in order to smooth possible intra-sample variance. PCR products were visualized on 1.5% agarose gels, then amplicon duplicates were pooled and purified using 0.8 × volumes of AMPure XP beads (Beckman Coulter). All the purified products were finally quantified with a Qubit Fluorometer (Thermo Fisher Scientific).

PCR indexing and normalization were based on the “16S Metagenomic Sequencing Library Preparation” protocol provided by Illumina, with the following major modifications: (1) PCR mixtures were in 25 μL final volume, using 2.5 μL of template DNA, 0.5 U of Phusion High-Fidelity DNA polymerase (Thermo Fisher Scientific), 1 × Phusion HF buffer, 200 μM of each dNTP, and 5 μL of each index primer. (2) PCR amplicons were normalized using a SequalPrep Normalization Plate (Thermo Fisher Scientific). Finally, amplicon libraries were equally pooled and sequenced using the Illumina MiSeq system (2 × 300 base pairs). The 16S amplicon sequences generated for this study can be found in the Sequence Reads Archive (SRA) at NCBI under the accession number PRJNA609227.

### Bioinformatic Pipeline

Sequenced reads from 16S amplicon libraries were analyzed with QIIME2 version 2019.7 (Bolyen et al., 2019). Denoising and amplicon sequence variants (ASVs) were identified using DADA2 (Callahan et al., 2016) QIIME2 plugin, trimming for low quality bases (forward reads at 290 bp length and reverse reads at 220 or 240 bp according to quality). Resulting ASVs were aligned with mafft aligner (Katoh et al., 2002) via q2-alignment plugin. The resulting multi-alignment was used to reconstruct phylogeny with fasttree2 (Price et al., 2010) via q2-phylogeny. Rarefaction curves were constructed using q2-diversity. Taxonomy was assigned to ASVs using the q2-feature-classifier plugin (Bokulich et al., 2018) that embeds a naïve Bayesian taxonomy classifier against SILVA database v 132 clustered at 99% identity (Quast et al., 2013).

### Statistics

Differences in the final (d4) community structure among sampling sites, sampling depths and treatments were tested with analysis of similarity (ANOSIM) and visualized with non-metric multidimensional scaling plots (NMDS). To estimate the amount of variance associated with individual factors (station, depth and treatment), permutational multivariate analysis of variance (PERMANOVA, function *adonis* in package *vegan*, Oksanen et al., 2019) with pairwise analysis was used. All permutations tests were considered significant at *p* < 0.05 with 9999 permutations. For the analyses related to beta diversity hypothesis testing, Bray-Curtis dissimilarity matrices were constructed using normalized read abundances (function *vegdist* in package *vegan*, Oksanen et al., 2019). Visualization of the microbial diversity data was done using normalized genera abundance, with the package *ggplot2* (Wickham et al., 2016).

The quality of 16S rRNA sequencing replicates was checked computing Spearman’s rank correlation coefficients between raw ASVs counts of experimental duplicates.

The compositional change in the prokaryotic community was investigated by calculating the average (across experimental duplicates) relative abundance (RA) change of each genus (deepest taxonomic resolution). ASVs that were not present at 1% RA as a minimum, in at least one sample, were pooled as “Others.” Changes in control samples were compared against the starting community (d0), whereas the effect of detrital particles amendments was compared against the controls. This comparison allowed to highlight both positive and negative responses of each genus to: (i) the enclosure itself and (ii) to the enrichments with phytodetritus.

To test if the composition of detrital particles (i.e., taxa composition within plankton samples) drove differential response in the number of attached prokaryotes, we fitted Generalized Linear Models (GLMs) using attached prokaryotes abundance as response variable and phytodetrital composition as independent variables. Models were fitted separately for each time point (i.e., d0, d1, and d4) using the function *glm.nb* embedded in the *MASS* package (Venables and Ripley, 2002). The Shapiro-Wilk test was used to check if residuals met the assumption of normal distribution.

The Mann-Whitney test was also used to detect statistically significant differences between control and amended microcosms at day 4 and to test for significant differences in variables among two or more factors. The Spearman’s rank correlation coefficient was calculated among selected variables. All the above tests were considered significant at *p* < 0.05 after correction for false discovery rate (fdr, Benjamini and Hochberg, 1995). All the statistical analyses were conducted in the R environment (v. 3.6.1, R Core Team, 2019).

## Results

### Methodological Considerations

The results of chemical analyses on the four detrital pools are summarized in Supplementary Table 1. To estimate the magnitude of organic C enrichments following the phytodetritus addition, we calculated the enrichment factors of DOC and POC in the amended bottles according to Hardy et al. (2005) with the formula:

$$\frac{{\left[XOC\right]}_{\left(added+environmental\right)}}{{\left[XOC\right]}_{environmental}}$$

Where [*XOC*]_(*added*+*environmental*)_ is the concentration of either DOC or POC in the amended bottles and [*XOC*]_(*environmental*)_ is the POC or DOC environmental concentration (i.e., before the amendments). Since the seawater was filtered onto 1 μm pore size prior to the detritus enrichments, we assumed that prokaryotic cells represented the only source of POC in the pre-amended microcosms. Thus, POC concentration was calculated converting prokaryotic abundance into carbon using the conversion factor of 13 fgC Cell^–1^ derived from Carlson et al. (1999) for prokaryotic cells in the Ross Sea. Environmental DOC concentrations in surface and bottom samples were provided by Relitti (unpublished data).

Enrichment factors (Supplementary Table 2) for DOC ranged between 1.0 and 1.1 and from 1.2 to 2.3 for the 1 and 10 μg L^–1^ treatments, respectively. Amendments with detritus resulted in consistently higher POC enrichment factors, ranging between 6.5 and 67.7 in 1 μg L^–1^ and between 56 and 906.3 in 10 μg L^–1^. These calculations highlight that POC enrichment factors following phytodetritus amendments were up to 2 orders of magnitude greater than DOC ones.

We are aware that some uncertainties in the metabolic rates and community composition might arise from the ambient pressure characterizing our incubations. Metabolic rates of bathypelagic prokaryotes have been shown to be overestimated at ambient pressure (Tamburini et al., 2013) while, from a community perspective, some evidence suggest that the effect might be group specific (La Cono et al., 2015). However, there still is a lack of general consensus about the effect of decompression on the metabolism of deep-sea microorganisms (Tamburini et al., 2013 and references therein). Our inferences consider both metabolic and community changes in relative terms, i.e., enhanced or suppressed by the detrital addition and/or composition. Hence, even if the absolute measured rates may be biased, our findings are likely not impaired by the depressurized incubation.

### Phytodetritus Composition

**FIGURE 1:**
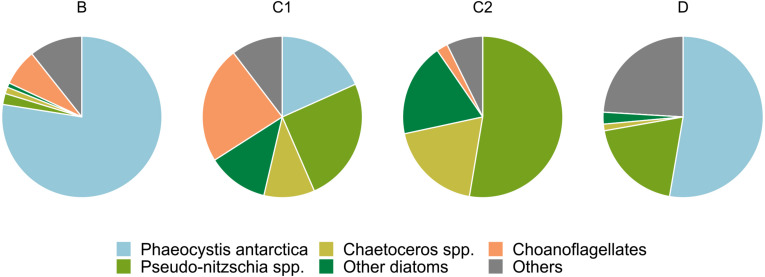
Compositional pie charts of plankton net samples used to generate phytodetritus.

The plankton net samples used to generate the phytodetritus showed marked compositional differences among stations (Figure 1). Plankton assemblages of stations B and D were dominated by *Phaeocystis antarctica*, accounting for 77 and 52% of the total pool, respectively. Diatom taxa were underrepresented in station B (4%), and accounted for 25% of microplankton cells at station D. The microplankton net sample of station C2 was instead dominated by diatom taxa, which accounted for 90% of the sampled community. Members of the *Pseudo-nitzschia* genus were the most abundant (52%), followed by the genus *Chaetoceros* (20%). A similar pattern was observed for C1 net samples, in which diatom taxa made up 50% of total pool. Representatives of the *Pseudo-nitzschia* genus were the most abundant (25%), followed by *Chaetoceros* spp. (10%). Choanoflagellates (23%) and *Phaeocystis antarctica* (18%) considerably contributed to the microplanktonic community retrieved at station C1.

### Viruses, Free-Living, and Particle-Attached Prokaryotes

Experiments B and C1 showed a low variability of VLPs abundance, which was remarkably constant over time and among treatments, with similar values in surface and bottom microcosms (Supplementary Figure 4). Bottles amended with detritus generated from the stations C2 and D showed a more variable temporal pattern, although rather similar among treatments (Supplementary Figure 4). In C2 surface bottles, both treatments resulted in a decreasing trend over time, even though abundance values were very similar to those in control bottles. Lower VLPs abundances were measured in bottom microcosms, which showed a rather similar decreasing trend over time, regardless of the treatment. An increase of VLPs over time was measured only in bottles amended with D detritus. A steep increase, deviating from the general pattern was evident on d4 in 10 μg L^–1^, resulting in the highest values measured in both surface and bottom enclosures (8.19 ± 3.48 and 5.24 ± 3.19 × 10^9^ VLPs L^–1^, respectively, Supplementary Figure 4).

Abundance of FL-HP (Supplementary Figure 4) showed a general increasing trend over time, more evident in 10 μg L^–1^ microcosms. Station B enclosures were a remarkable exception to this pattern, with FL-HP abundance extremely constant across time, treatment and depth (on average, 0.10 ± 0.01 × 10^9^ cells L^–1^, Supplementary Figure 4). In C1 and C2 bottles, the effect of the detritus addition became evident only on d4, whereas between d0 and d2, FL-HP abundances were very similar to those in control samples. The addition of detritus at 10 μg L^–1^ resulted in a steep increase of FL-HP abundance in surface C1 samples (approximately 5 times higher than control, Supplementary Figure 4), whereas in bottom samples FL-HP abundance was not clearly affected by the particles enrichments. Station C2 enclosures showed an inverse pattern, with a stronger response of bottom samples to particles addition (∼6 times higher than control on d4). The highest cell numbers were measured in D samples in both surface (5.49 ± 0.35 × 10^9^ cells L^–1^) and bottom samples (1.92 ± 0.83 × 10^9^ cells L^–1^). Addition of 1 μg Chl *a* L^–1^ equivalent in surface samples had mild effect on FL-HP abundance, whereas the strongest amendment yielded a steep increase of FL-HP cells in both surface and bottom samples (Supplementary Figure 2).

**FIGURE 2:**
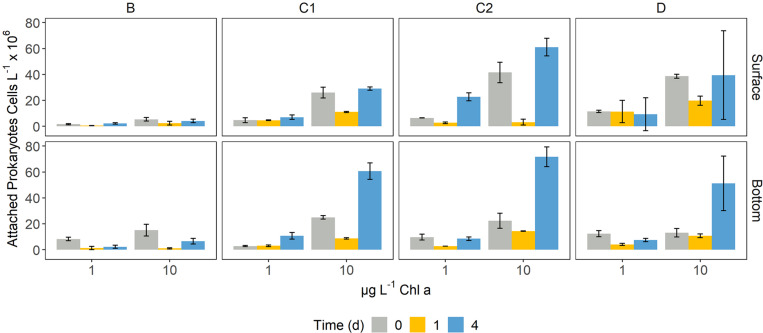
Bar plots showing the abundance of attached prokaryotes on d0, d1, and d4 in each of the microcosms. Error bars represent the standard deviation of two experimental replicates.

The abundance of PA ranged between 0.49 ± 0.09 and 71.71 ± 7.67 × 10^6^ cells L^–1^ (Figure 2). The lowest number of attached cells was measured on d0 in B surface bottles amended with 1 μg Chl *a* L^–1^, while the highest number of PA cells was found on d4 in C2 bottom samples with 10 μg L^–1^. Over time, PA abundance was constant or decreasing between d0 and d1, followed by an increase on d4 (Figure 2). B experiments showed the lowest abundance of particle-attached prokaryotes, in both surface and bottom microcosms, whereas station C2 presented the highest PA abundance. PA abundance on d0 was higher in surface enclosures, except for B bottles, where the initial PA cells number was higher in bottom samples (Figure 2). On d4, bottom microcosms presented a higher PA abundance than surface ones, except for C2 samples (Figure 2).

Generalized linear models identified a significant dependence of the number of attached prokaryotes on the taxa composing the phytodetrital pool (Supplementary Table 3), with a varying temporal pattern. Attached cells abundance on d0 was found significantly related to *Pseudo-nitzschia* spp. abundance (*p* < 0.05). On d4, three of the four considered groups (i.e., *Pseudo-nitzschia* spp., *Phaeocystis antarctica*, *Chaetoceros* spp.) drove a significant response of the PA cells abundance (*p* < 0.001). The GLM fitted with data from d1 did not meet the assumption of normal distribution of the residual terms (Shapiro−Wilk test, *p* < 0.05) and was therefore not further considered.

### Microbial Metabolic Activity

#### Exoenzymatic Activities

**FIGURE 3:**
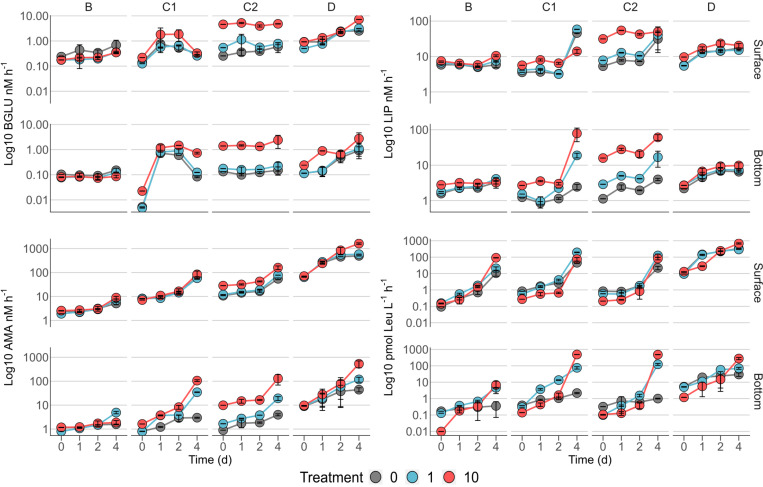
Time courses of β-glucosidase (BGLU), lipase (LIP), leucine-aminopeptidase activities (AMA), and heterotrophic carbon production (HCP). 0, controls; 1, 1 μg L^–1^ Chl *a*; and 10, 10 μg L^–1^ Chl *a*. To ease comparison, metabolic rates are plotted on Log *Y*-axes, which are differentially scaled. Error bars represent the standard deviation of two experimental replicates; where not visible, they are embedded in the symbol.

Minima of the three EEAs were all measured in bottom C1 bottles (BGLU: 0.01 ± 0.00 nM h^–1^; AMA: 0.79 ± 0.08 nM h^–1^; and LIP: 0.86 ± 0.07 nM h^–1^; Figure 3). Maxima of BGLU and AMA were measured in surface bottles amended with station D phytodetritus (7.19 ± 0.00 and 1622.24 ± 113.55 nM h^–1^, respectively, Figure 3), while the fastest lipid degradation was found in bottom microcosms supplemented with C1 detrital particles (78.61 ± 32.65 nM h^–1^, Figure 3).

Protease activity showed a general increasing trend in all the experiments, with steeper slopes between days 2 and 4 (Figure 3). Slower rates were observed in B bottles, while maxima were measured in bottles amended with particles from station D (Figure 3). Treatments at 10 μg L^–1^ yielded the strongest increase in peptidase activity in bottom bottles enriched with detritus from stations C1, C2, and D compared to controls on d4 (approximately 35, 30 and 10 times, respectively, Figure 3). The amendment of B bottom microcosms with 1 μg L^–1^ sustained a ∼3 times higher AMA activity compared to control and 10 μg L^–1^ treatment (Figure 3).

Lipid degradation was slower in experiments B and D in both surface and bottom bottles. Experiments C1 and C2 showed the highest values of lipase activity in bottom microcosms, where the maximum value was measured (experiment C1, Figure 3). A mild effect of the detritus enrichments was visible in surface B and D bottles, while bottom experiments were not clearly affected by particle amendments (Figure 3). The enrichment with 10 μg L^–1^ in surface C1 bottles resulted in a ∼4 times lower rates compared to control and 1 μg L^–1^ at day 4 (Figure 3). In bottom C1 bottles, the highest Chl *a* amendment yielded an increase in LIP activity of approximately 40 and 15 times compared to control and 1 μg L^–1^, respectively (Figure 3). A similar trend was observed for C2 experiments, although with a milder increase (Figure 3). Surface C2 bottles were characterized by higher LIP rates in the 10 μg L^–1^ throughout the experiment, compared to control and 1 μg L^–1^ (Figure 3).

Carbohydrate hydrolysis showed a peculiar response in each of the experiments (i.e., B, C1, C2, and D), highly conserved in both surface and bottom bottles. The lowest BGLU activity was measured in station B microcosms. In station C1 experiments, enrichments at 10 μg L^–1^ induced slightly higher BGLU rates compared to control and 1 μg L^–1^. The general pattern, showing a steep increase in the first 24 h and then a drop to initial values on d4, was common to all the experimental conditions (Figure 3). Amendments with C2 detritus (Figure 3) yielded the strongest response in the 10 μg L^–1^ treatment, with rates ∼8 and 17 times higher than 1 μg L^–1^ treatment and control, respectively. An increasing trend over time was measured in both surface and bottom D bottles. While the addition of detrital particles at 1 μg L^–1^ did not produce a clear effect on glycolytic rates (Figure 3), enrichments at 10 μg L^–1^ resulted in a steeper increase of BGLU activity on day 4, which was approximately 3 times higher than control and 1 μg L^–1^ in both surface and bottom bottles (Figure 3).

#### Heterotrophic C Production and DDIC Uptake

Heterotrophic carbon production (Figure 3) showed an overall strong response to detritus enrichment, especially on d4. Rates of prokaryotic production spanned over 4 orders of magnitude, ranging between 0.01 ± 0 and 682.83 ± 57.66 pmol Leu L^–1^ h^–1^. Range extrema were measured at the highest Chl *a* concentration (10 μg L^–1^) in bottom B bottles and surface D bottles, respectively (Figure 3). As observed for the EEAs, overall slower prokaryotic production rates were observed in B experiments, with higher values in surface bottles. Amendments with detritus yielded the strongest response at 10 μg L^–1^, with HCP rates approximately 9 and 16 times higher than control in surface and bottom bottles, respectively (Figure 3). Enrichment at 1 μg L^–1^ in experiment C1 resulted in a steep increase of prokaryotic production on d4, with rates ∼4 and 3 times higher than control and 10 μg L^–1^, respectively (Figure 3). In bottom C1 bottles, HCP rates strongly increased in response to 10 μg L^–1^ amendments, peaking at 489.00 ± 6.29 pmol Leu L^–1^ h^–1^, the maximum value measured in bottom samples (Figure 3). Experiment C2 showed a similar response in both surface and bottom bottles (Figure 3). Experiment D microcosms showed a steady increasing trend of Leucine incorporation rates over time. The detritus enrichment induced the strongest response at the highest concentration (10 μg L^–1^), resulting in HCP rates approximately 3 and 4 times higher than control in surface and bottom bottles, respectively (Figure 3).

DDIC is an often neglected channeling of environmental C into prokaryotes and can be associated both to heterotrophic and autotrophic metabolism (see section “Detritus-Induced Functional Changes”) The enrichments with detritus at the lowest concentration stimulated dark DIC uptake in C1 and C2 enclosures, with a stronger effect on the latter (∼2 times higher than control, Table 2), while negatively affecting D and B enclosures (Table 2). The enrichments at 10 μg L^–1^ strongly enhanced DDIC rates in station D, C1 and C2 (approximately 10, 5, and 6 times higher than control, respectively), while had a slight negative effect on B microcosms (Table 2). Bottom DDIC uptake rates were unaffected by the particle amendments at 1 μg L^–1^, while the strongest enrichments yielded a remarkable enhancement of dark DIC uptake in B and C2 bottles (∼2 and 6 times relative to controls, respectively).

**TABLE 2:** Dark dissolved inorganic carbon (DDIC, ngC L^–1^ d^–1^) uptake rates in control and amended microcosms measured on d0 (see section “Microbial Metabolic Activities”).

Depth	Treatment	B	C1	C2	D
Surface	Control	2.60 ± 0.19	0.40 ± 0.00	14.39 ± 4.95	13.26 ± 2.90
	1 μg L^–1^ Chl *a*	1.91 ± 0.77	1.40 ± 1.42	31.88 ± 8.43	3.69 ± 1.74
	10 μg L^–1^ Chl *a*	1.64 ± 0.19	2.00 ± 0.57	94.23 ± 47.84	166.41 ± 1.74
Bottom	Control	2.41 ± 1.80	3.94 ± 2.05	6.71 ± 3.42	8.49 ± 0.00
	1 μg L^–1^ Chl *a*	1.42 ± 0.40	3.74 ± 0.59	10.20 ± 4.55	10.19 ± 3.20
	10 μg L^–1^ Chl *a*	4.67 ± 0.20	3.11 ± 0.88	41.73 ± 20.30	2.97 ± 1.40

*Results are presented as mean ± SD of two experimental replicates.*

### Prokaryotic Diversity and Community Composition

Following the bioinformatic pipeline, the 16S rRNA dataset comprised 1950252 reads from the 56 analyzed samples, with, on average, 34825.92 reads per sample, totaling 420 prokaryotic ASVs. The lowest number of reads was found in the initial (d0) C2 surface sample (*n* = 12110), while sample replicate 2 of bottom D sample amended with 10 μg L^–1^ scored the highest value (*n* = 81610). The whole prokaryotic diversity was captured by the chosen sequencing effort, as shown by the rarefaction curves (Supplementary Figure 5). ASVs richness (i.e., number of unique ASVs retrieved) ranged between 43 and 219. Both values were measured in C2 bottom enclosures, in replicate 2 of the 10 μg L^–1^ amendment on d4 and d0, respectively. A general decreasing trend in richness (Supplementary Figure 6) was observed with increasing Chl *a* concentration (i.e., 0, 1 and 10 μg L^–1^), particularly evident in bottom samples, whose initial communities were characterized by a significantly higher richness compared to surface ones (Mann–Whitney, *p* <0.05). Segregation by depth did not reveal significant differences in richness between stations (Mann–Whitney, *p* >0.05). The sequencing duplicates were highly consistent, being significantly correlated at *p* < 0.001, with Spearman’s rho values ranging between 0.60 and 0.90 (Supplementary Figure 7).

**FIGURE 4:**
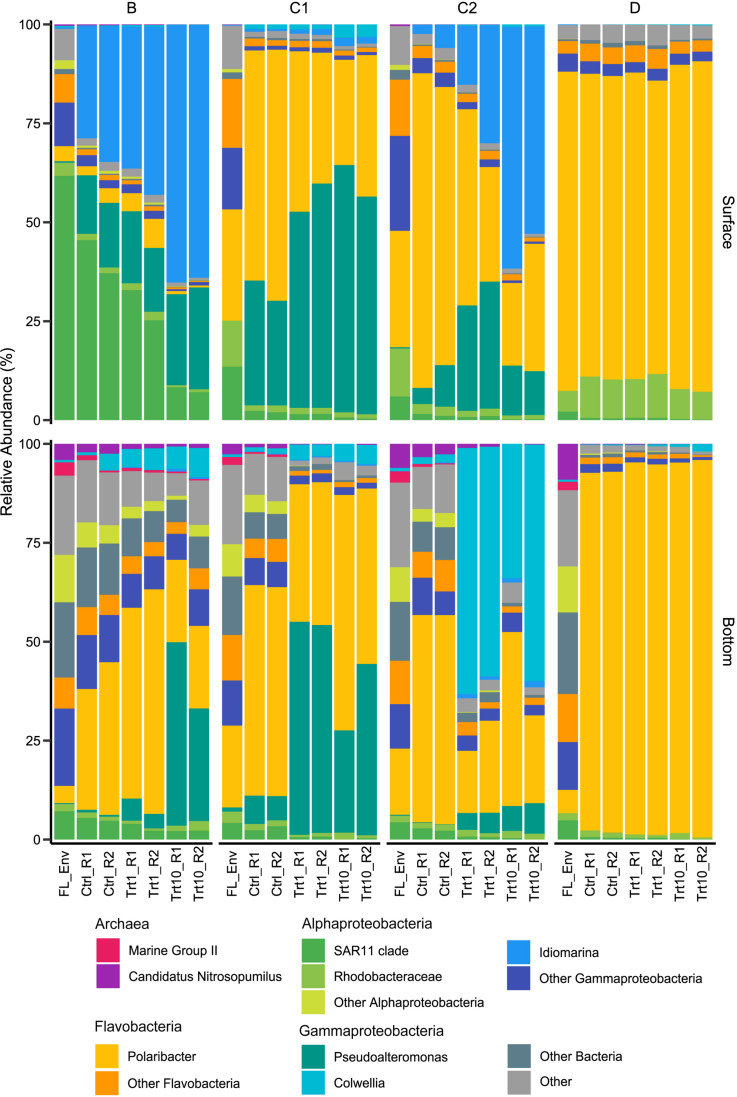
Relative abundance plots of major taxa (>1% in at least one sample). FL_Env, initial free-living (1 μm-filtered) community, Ctrl, control samples; Trt1, amendments at 1 μg L^–1^ Chl *a*; Trt10, amendments at 10 μg L^–1^ Chl *a*; R1 and R2 identify the two experimental replicates. A detailed version of the figure with all subgroups showed is available in the Supplementary Material.

In terms of initial community composition, the relative abundance of major taxa (RA > 1%, Figure 4 and Supplementary Figure 8) significantly varied with depth (ANOSIM *R* = 0.83, *p* < 0.05). Surface samples of station B and D were respectively dominated by ASVs mapping to the SAR11 clade (61.85%) and to the *Polaribacter* genus (75.99%). The latter represented the most abundant taxon in stations C1 and C2 too (22.68 and 24.87%, respectively), sided by SAR11 ASVs at station C1 (13.50%) and by members of the Gammaproteobacterial family *Nitrincolaceae* at station C2 (13.45%). Initial bottom communities (Figure 4 and Supplementary Figure 8) were dominated by members of the phyla *Proteobacteria* (30.15 ± 6.32%) and *Bacteroidetes* (24.54 ± 9.55%). The former characterized the communities of stations B and D (39.47 and 28.77%, respectively), whereas stations C1 and C2 were dominated by the latter (34.97 and 29.66%, respectively). Classes *Alpha*- and *Gamma-proteobacteria* accounted for most of the Proteobacterial sequences (11.53 ± 1.84 and 14.74 ± 3.78%, respectively) in these stations, while most of the *Bacteroidetes* abundance was made up by *Flavobacteriales* (22.82 ± 9.16%). Among the *Alpha-proteobacteria*, members of the SAR11 clade represented approximately half of the retrieved sequences (5.03 ± 1.55%), with the highest relative abundance observed in station B samples (7.30%). *Gamma-proteobacteria* were mostly represented by sequences assigned to the family *Thiomicrospirales*, especially abundant at station B (10.69%, Supplementary Figure 8), and *Oceanospirillales* (on average, 4.69 ± 4.02 and 4.27 ± 0.73%, respectively, Supplementary Figure 8). Archaeal ASVs, belonging to Marine Group II and *Nitrosopumilaceae*, represented, on average, 8.08 ± 2.71% of the bottom initial community (Figure 4 and Supplementary Figure 8).

**FIGURE 5:**
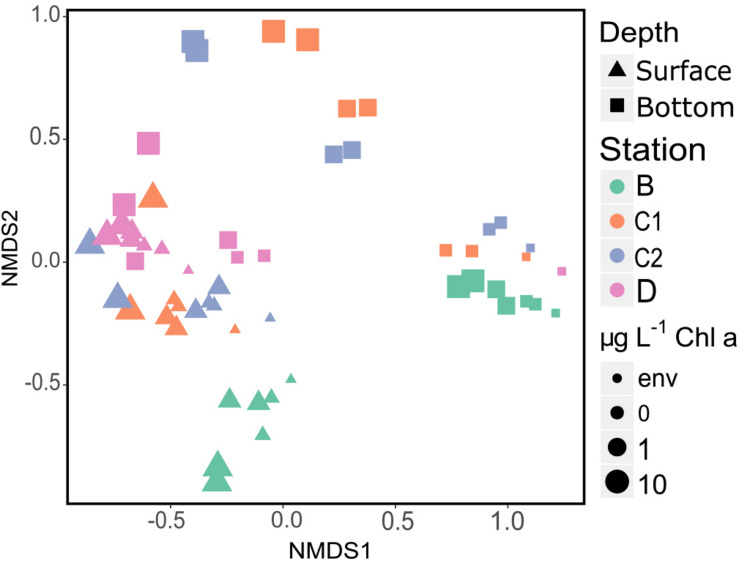
NMDS plot (stress = 0.11) showing Bray–Curtis dissimilarity in community composition. Samples are color, shape and size coded according to sampling site, sampling depth and treatment, respectively (see legend). Env, initial community d0, 1 μm-filtered; s, surface; b, bottom. Both replicates for each sample are plotted.

NMDS plot of the final prokaryotic communities showed that samples separated well according to sampling station and depth (Figure 5). The highest amount of variance was explained by the sampling site (40%, Table 3), highlighting both the effect of the “seed” community and of the addition of different kinds of detrital particles on the observed differences.

**TABLE 3:** Results of permutational multivariant analysis of variance (PERMANOVA) of final prokaryotic communities based on Bray-Curtis dissimilarities of read relative abundance.

Factor	d.f.	SS	pseudo F	R^2^
Sampling Site	3	4.4	14.65	0.40*
Sampling Depth	1	1.87	18.72	0.17*
Treatment	2	0.63	3.15	0.05
Residuals	48	4.11	–	0.37
Total	55	11.03	–	1

The factor “Sampling Site” represents both the effect of the “seed” community and of the amendment with the site-specific detrital pool. d.f., degrees of freedom; SS, sum of squares. *Denotes significance at p < 0.001, calculated after 9999 permutations.

**FIGURE 6:**
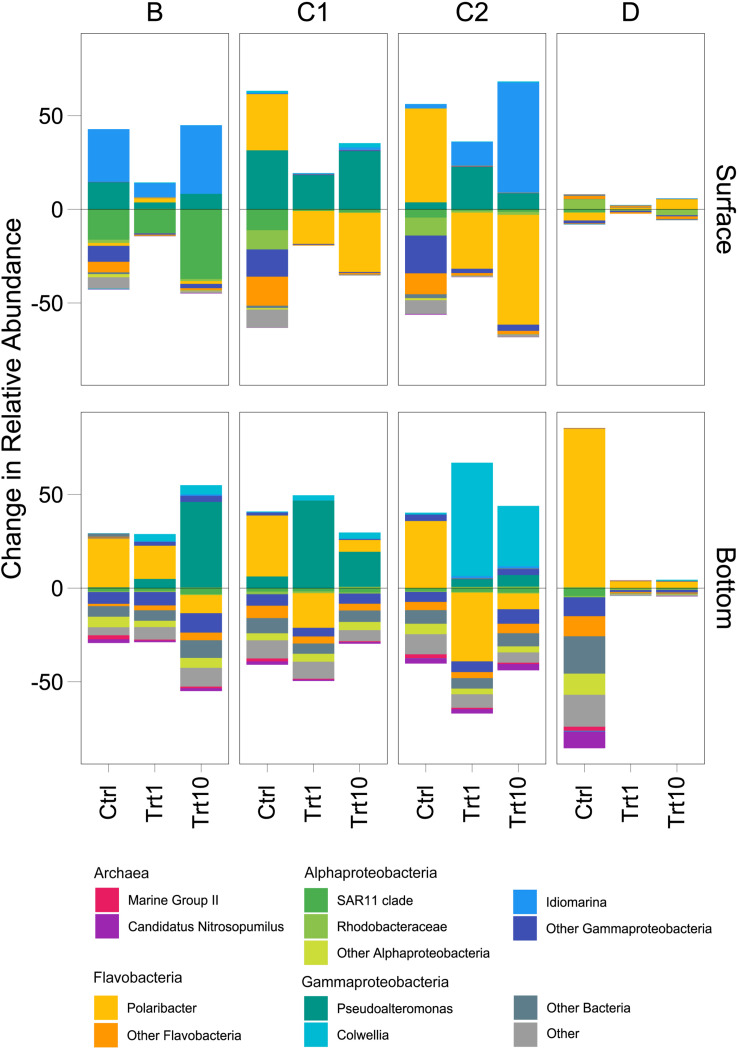
Treatment-related shifts in prokaryotic community. Shifts in controls are compared to the initial (d0, 1 μm-filtered) community, whereas taxa shifts in treatments (i.e., 1 and 10 μg L^–1^) are compared against the controls (both on d4). Ctrl, control samples; Trt1, amendments at 1 μg L^–1^ Chl *a*; Trt10, amendments at 10 μg L^–1^ Chl *a.* A detailed version of the figure with all subgroups showed is available in the Supplementary Material.

The enclosure of the initial prokaryotic communities led to an overall increase in the relative abundance of members of the *Flavobacteriaceae* family, mostly due to ASVs mapping to *Polaribacter* genus (Figure 6 and Supplementary Figure 9). Station B represented an exception to this pattern, with changes in relative abundance mostly due to the genera *Idiomarina* (Alteromonadales) in surface samples and *Polaribacter* (Flavobacteriales) in bottom ones (Figure 6). Community changes in samples amended with detrital particles were mainly driven by the increase of the relative abundance of members of the order Alteromonadales to the detriment of Flavobacteriales representatives (Figure 6). Distinct Alteromonadales genera characterized the observed changes across sampling sites and depths. The genus *Idiomarina* increased in surface B and C2 enriched samples, particularly in the 10 μg L^–1^ Chl *a* treatment, while the genus *Colwellia* was largely responsible for the changes observed in amended C2 bottom samples (Figure 6). The genus *Pseudoalteromonas* was positively affected by the detritus addition in both surface and bottom samples of station C1 and in bottom B bottles (Figure 6). Surface D enclosures showed very few changes in relative abundance in response to both the enclosure and to the amendment with phytodetritus (Figure 6). The enclosure of bottom D samples strongly selected for *Polaribacter* ASVs, with relative abundance increasing ∼ 40 times compared to environmental samples (Figure 6). At station D the amendments with detrital particles induced only minor community changes in both surface and bottom samples.

## Discussion

### Detritus-Induced Changes in Prokaryotic Abundance and Community Structure

**FIGURE 7:**
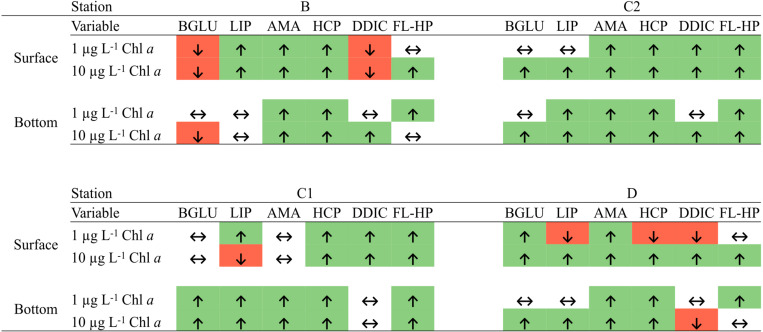
Significant differences between control and amended microcosm at day 4 (Mann–Whitney test, *p* < 0.05). ↑, enhanced by phytodetritus; ↓, inhibited by phytodetritus; ↔, no significant variation detected. BGLU, β-glucosidase activity; LIP, lipase activity; AMA, leucine-aminopeptidase activity; HCP, heterotrophic carbon production; DDIC, dark dissolved inorganic C uptake rates; FL-HP, free-living heterotrophic prokaryotes abundance.

Our analyses identified sampling site and depth as the main driving factors explaining the observed differences in final (d4) communities (see section “Microbial Metabolic Activity” and Figure 5). The addition of “small” amount of detritus (1 μg L^–1^ Chl *a* equivalent) was enough to select for the growth of specific prokaryotic taxa within the *in situ* free-living communities In fact, we did not find a significant effect of phytodetritus concentration (i.e., 1 vs. 10 μg L^–1^, Table 3) on the final prokaryotic communities, suggesting that the major drivers of the observed changes were the features of the phytodetrital particles rather than their concentration. These results suggest that the interaction of the initial free-living assemblage with the particulate detrital pool resulted in site-specific changes, differentially affecting surface and bottom assemblages. Enrichments with *Phaeocystis*-derived detrital particles yielded significantly higher FL-HP abundance in surface samples amended with 10 μg L^–1^ and in bottom bottles amended with 1 μg L^–1^ (stations B and D, Figure 7). This pattern suggests that, in surface samples, mild detritus enrichments induced a community-level response (i.e., shifts in community structure with constant cell abundance, Reintjes et al., 2019) to the POM features, while stronger enrichments select for specific fast-responsive taxa (i.e., Alteromonadales in B and Flavobacteriales in D, Figure 6). It is noteworthy that bottom samples showed an exact inverse pattern (Figure 7), highlighting that small pulses of organic matter are rapidly exploited by conditionally rare copiotrophs, while more consistent loads of POM re-shuffle the whole community. At stations C1 and C2, enrichments with diatom-derived detritus yielded significantly higher FL-HP abundance than control samples. The increase in FL-HP in those samples is coupled with the marked increase in the relative abundance of *Pseudoalteromonas* and *Idiomarina* genera, respectively (Figure 6), suggesting that the changes in community composition were the result of the emergence of these genera rather than a community-level response to the detritus addition.

Starting communities (Figure 4 and Supplementary Figure 8) comprised typical free-living lineages as SAR11, SAR86, and AEGEAN-169 (Dupont et al., 2012; Giovannoni et al., 2014) sided by clades often observed associated with phytoplankton blooms or reported as feeders on algal-derived compounds (e.g., NS3 and NS5 marine groups and Rhodobacteraceae; Halsey et al., 2012; Amin et al., 2015; Teeling et al., 2016). The latter were more represented in bottom initial communities, suggesting that, albeit free-living, those communities harbored the potential to find, colonize and degrade phytodetrital particles. Starting bottom assemblages were compositionally similar, as expected, since they were collected within the same deep-water mass (High Salinity Shelf Water). Indeed, in the same area, in previous studies we had observed a segregation of deep prokaryotic (Celussi et al., 2010) and protist (Zoccarato et al., 2016) communities according to the water mass they were sampled in. On the other hand, due to the strong physical and biological spatial gradients of the upper water column (Rivaro et al., 2019), surface initial communities widely varied across sampling site (Figure 5). Flavobacteria and SAR11 clades drove the observed differences, accounting for the 75.99 and 61.55% of the sequences retrieved in D and B, respectively (Figure 4).

Flavobacteria are well-known degraders of complex organic molecules, such as proteins and polysaccharides, being less effective in the uptake of smaller and simpler organic substrates (Wietz et al., 2015; Fourquez et al., 2016). These experimentally observed traits are supported by comparative genomic studies showing a conspicuous repertoire of genes involved in high molecular weight (HMW) compounds transport and degradation, sided by a lower number of genes devoted to handle simpler organic substrates (González et al., 2008; Gómez-Pereira et al., 2012; Fernández-Gómez et al., 2013; Williams et al., 2013). It is, therefore, not surprising that Flavobacteria, mainly represented by the *Polaribacter* genus, constituted a significant fraction of the microcosms community (except for B surface enclosures, Figure 4 and Supplementary Figure 8). At the time of sampling, a diatom bloom was in place in the station D surface layer (Supplementary Results), thus explaining the dominance of Flavobacterial ASVs in this sample. Indeed, most of those ASVs mapped to the genus *Polaribacter*, a taxon reported as associated with diatom-derived organic matter in both environmental surveys (Teeling et al., 2012, 2016) and experimental enclosures (Wietz et al., 2015; Landa et al., 2018). Noteworthy, amendments with detrital particles at both concentrations in station D samples showed very little community changes (Figure 6 and Supplementary Figure 9). While this was expected in surface samples, as the initial community (i.e., 1 μm-filtered) was already dominated by Polaribacter ASVs (Figure 4), the fact that bottom samples showed the same dynamics, in the face of a highly diverse initial community (Figure 6 and Supplementary Figure 9), was rather puzzling. Although we cannot rule out the influence of the bottle effect, one possible explanation relies on the phytodetrital composition which, besides the high number of diatoms, was mainly composed by *Phaeocystis antarctica* (>50%, Figure 1). Colonial forms of *P. antarctica* allocate ∼50% of carbon production to the extracellular mucus that makes up the scaffold of the colonial matrix (DiTullio et al., 2000), making the *Phaeocystis*-derived detritus a source of HMW polysaccharides. Previous enrichment experiments with *Phaeocystis*-derived material have shown how the degradation of this organic pool is mostly carried out by Flavobacteria (Brussaard et al., 2005), further explaining their dominance in D samples. Flavobacterial ASVs, mostly represented by the *Polaribacter* genus, contributed to a consistent part of the final prokaryotic community in all bottom unamended microcosms (Figure 4 and Supplementary Figure 8). Recent *in vivo* and *in vitro* studies (Reintjes et al., 2017, 2019) have demonstrated that Flavobacteria representatives are able to assimilate large polysaccharides in their periplasmic space, where subsequent hydrolysis and uptake processes take place. This mechanism, defined as “selfish” uptake, is highly competitive as it implies a reduced diffusive loss of hydrolysis products. The dominance of Flavobacterial representatives in control samples is therefore explained by their capability to efficiently utilize HMW DOM, outcompeting other members of the community. Previous experiments with organic matter enrichments showed similar results (Wietz et al., 2015; Luria et al., 2017; Landa et al., 2018), confirming the ability of this group to efficiently utilize HMW organic matter.

SAR11 is a diverse group, widespread in oligotrophic water across the global ocean (Giovannoni, 2017). Genomic analysis on both environmental and cultivated members of this clade have pointed out peculiar adaptations to thrive in nutrient-poor environments (Sowell et al., 2009; Thompson et al., 2013). This lineage is not able to degrade HMW organic matter, supplying this lack with a high abundance of LMW organic matter transporters (i.e., amino-acids and sugars, Tripp, 2013). Although the relative abundance of SAR11 clade members decreased with the addition of detrital particles, it represented a consistent fraction in all samples (>1%, Figure 4), showing a higher occurrence in samples where Flavobacterial ASVs were poorly represented (e.g., station B experiments, Figure 4). Within their conceptual model, Arnosti et al. (2018) put out a third player, the “scavenging” bacteria, not able to degrade HMW organic matter on their own but rather “scavenging” hydrolysis products produced by external degraders. The SAR11 clade remarkably fits this role, and its “scavenging” behavior has been confirmed in their *in vivo* experiments. Our results confirm these findings, linking the presence of SAR11 to the prevalence of external hydrolysis of organic matter (as suggested by the high RA of Alteromonadales, Figure 4) and further suggest that, under the appropriate conditions, “scavenging” behavior may be more effective than “selfish” hydrolysis.

While both SAR11 and Flavobacteria were well represented in the initial prokaryotic community, ASVs belonging to the Gammaproteobacterial family Alteromonadales were initially rare, yet they became prevalent in all the enriched bottles of station B, C1 and C2 (Figure 6). A similar pattern has been previously observed in HMW organic matter amendment experiments (Luria et al., 2017; Balmonte et al., 2019) and all these findings point out a major role of these conditionally rare, copiotroph taxa in exploiting organic matter pulses. The increase in Alteromonadales ASVs was always at the detriment of Flavobacterial representatives (Figure 6), in agreement with what has been observed in DOM enrichment experiments in the Southern Ocean (Luria et al., 2017; Landa et al., 2018). Alteromonadales can rapidly take advantages of transient sources of organic matter through their flexible metabolism, which allow them to utilize a wide array of phytoplankton-derived organic molecules (Liu et al., 2017; Sperling et al., 2017). These characteristics make this family the archetype of the r-strategist (Pedler et al., 2014), explaining its dominance in the amended samples. It is interesting to note that within the Alteromonadales order, three different genera dominated in different groups of experiments: *Pseudoalteromonas* and *Idiomarina* co-occurred in B and C1 samples and in C2 surface samples, whereas the genus *Colwellia* was dominant in C2 bottom enclosures (Figures 4, 6). All these three genera have been shown to be able to degrade dimethylsulfoniopropionate (DMSP), a sulfur-containing molecule produced by phytoplankton (Zeng, 2019). As DMSP is mainly produced by diatoms and *Phaeocystis* spp., the ability to degrade this compound may have been a prominent factor driving the observed Alteromonadales prevalence in amended samples. Among the three genera, *Pseudoalteromonas* is the only one able to grow using DMSP as a sole source of carbon (Zeng, 2019), a characteristic that may explain its exclusive dominance in samples amended with C1 detritus, the particles pool of which was almost exclusively composed by diatoms (Figure 1). In agreement with our results, specific taxa within this bacterial family have been observed to success during different stages of phytoplankton blooms, indicating (*i*) that these taxa possess the hydrolytic machinery necessary to rapidly process HMW organic matter and (*ii*) that different organic matter source drives specific community changes (Teeling et al., 2012, 2016; Buchan et al., 2014).

A conspicuous amount of Archaeal ASVs was retrieved in bottom samples at all stations (∼10%, Figure 4). Albeit by the end of the experiment most of the Archaeal community was wiped out by bacterial ASVs, their presence throughout the incubation was noteworthy (∼1% on d4, Figure 4). Most of the Archaeal ASVs retrieved in our samples belonged to members of Marine Group II (MG II, Euryarchaeota), a group identified as a consistent member of prokaryotic community in both temperate and polar oceans (Zhang et al., 2015; Quero et al., 2020). Metagenomic-inferred physiology suggest that members of MG II possess abundant genes deputed to the handling and utilization of HMW organic matter (Deschamps et al., 2014). Consistent with these findings, Orsi et al. (2015) found an enrichment of MGII sequences in the particle-attached fraction, indicating physical association with particles and therefore, the presence of detrital particles in our experiments may have provided MG II with exploitable substrates, granting their persistence throughout the incubation period.

The particle type, taken as a proxy of the organic matter quality, has been suggested to play a key role in the processes governing the activity and composition of the particle attached bacterial community (Grossart et al., 2005; Teeling et al., 2012). Indeed, we found significant differences in the number of PA prokaryotes according to the detrital pool supplied, with diatom-based detritus yielding the highest number of associated bacteria on d4 (Figure 2). Albeit compositionally different, the final communities were rather similar in terms of potential interaction with particles, harboring two of the major players in particles colonization and degradation (i.e., Flavobacteriales and Alteromonadales). The results of the GLMs showed that the abundance of *Pseudo-nitzschia* was the only significant factor driving the number of attached bacteria on d0 (*p* < 0.05), whereas on d4 the response of PA bacteria was significantly related to all the other microplankton taxa (*p* < 0.001 for *Pseudo-nitzschia* and *Phaeocystis spp.*, *p* < 0.05 for *Chaetoceros spp.*, Supplementary Table 3). Given these results, we hypothesize that the observed increase of attached prokaryotes over time may be due to the intrinsic properties of the detrital pool supplied. Diatom-derived detritus indeed, may have represented a more suitable colonization substrate than *Phaeocystis*-dominated one, providing more particulate material per unit Chl *a* (Supplementary Table 1). The utterly complex shape of diatom-derived aggregates may enhance prokaryotic colonization by making available a wide variety of microniches as well as by enhancing coagulation with other particles (Zetsche et al., 2020). These properties likely explain the higher number of attached cells that we found on diatom-containing detrital pools. Furthermore, we found significant positive correlation between viruses and diatom abundance in net samples [Spearman’s rho: *Pseudo-nitzschia* spp. = 0.31; *Chaetoceros* spp. = 0.19; other diatoms = 0.19, *p* (fdr) <0.01, *n* = 128], suggesting that especially diatom-derived phytodetritus represented hot-spots of viral activity during the incubations. This intense activity could thus have caused lysis-derived DOM to diffuse from the aggregates, increasing particles detectability by chemotactic prokaryotes and thus the PA abundance (Kiørboe and Jackson, 2001; Riemann and Grossart, 2008). In addition, this DOM may have represented an additional source of organic matter for FL-HP, contributing to their significant increase in amended samples relative to control ones (Figure 7). It must be noted, however, that the abundance of virus-like-particles was remarkably constant in our microcosms, except for bottles amended with detritus D (Supplementary Figure 4). This suggests a dynamic balance between production and loss processes, possibly represented by detritus-enhanced prokaryotic activity (and thus viral production) vs. detritus-enhanced viral adsorption by particles (Fuhrman, 1999). Despite the similar detrital composition, Stations D and B showed a remarkable difference in colonization yield (Figure 2). Shifts in microbial community composition as the particle ages or sink are correlated with changes in community functioning (Fontanez et al., 2015), leading in turn to modifications of the nutritional properties of the particles (Smith et al., 1992; Martinez et al., 1996). Moreover, the process of particle colonization is highly dynamic (Kiørboe et al., 2004), thus if the particle-OM labile pool is exhausted or selective degradation reduces its palatability for the resident prokaryotes, the most cost-effective strategy consists in finding another particle to exploit, thus increasing the probability to find free-living cells. Furthermore, albeit both detrital pools were *Phaeocystis*-dominated, a consistent fraction of station D detritus was composed by diatoms (Figure 1), which contain more particles (i.e., active surfaces) per Chl *a* unit than *Phaeocystis* (DiTullio and Smith, 1996; Supplementary Table 1), thus providing prokaryotes with a physical scaffold for colonization.

### Detritus-Induced Functional Changes

In terms of functional response to phytodetritus addition (Figure 7), diatom-derived organic matter mostly exerted a positive effect on metabolic rates, whereas when enriched with *Phaeocystis*-derived detritus, microbial communities showed shifts in organic matter degradation patterns.

Metabolic rates measured in microcosms amended with B detritus were consistently the lowest among the four groups of samples (Figure 3). This may be explained by two hypotheses, regarding (*i*) the composition of the supplied detritus and (*ii*) the native prokaryotic community composition. The detritus supplied to B bottles was mainly composed by *Phaeocystis antarctica*, which allocates a significant fraction of its carbon content to carbohydrates (Mathot et al., 2000) that are stored in the forms of glucan or polysaccharides as the growth progress toward senescence (Alderkamp et al., 2007). Our measurement of carbohydrate degradation is limited to the activity of β-glucosidase and thus it is possible that, while this enzyme showed a reduced activity following the addition of detrital particles, a whole, untested, set of polysaccharide degrading enzymes may have been produced to cope with the complexity of the organic matter in this detrital pool. Recent experiments have demonstrated how, following the addition of HMW organic matter, many different glycolytic enzymes are produced (Balmonte et al., 2019), corroborating our hypothesis. Culture and mesocosm-based experiments have shown that Flavobacteria was the major bacterial group involved in *Phaeocystis*-derived organic matter processing (Brussaard et al., 2005; Alderkamp et al., 2006) and thus their low proportion in B enclosures may explain the low exoenzymatic activities. Consistent with this hypothesis, we observed a shift from carbohydrate to lipid degradation in surface samples (Figure 7). This observation was coupled with the increase in RA of *Idiomarina* in amended samples. It has been reported that the genome of *Idiomarina* shows a higher proportion of lipid metabolism-related genes compared to substantial loss of sugar metabolism genes (Hou et al., 2004). The enhancement of BGLU rates in D experiments, despite the similar detrital composition, further corroborate our hypothesis, linking *Phaeocystis*-derived organic matter degradation with a Flavobacteria-dominated community (Figures 4, 7).

DDIC fixation rates were differentially affected by the distinct pools of phytodetrital particles supplied (Figure 7 and Table 2). Diatom-derived detritus significantly enhanced DDIC uptake, congruently with the other measured metabolic rates, whereas samples amended with *Phaeocystis*-dominated phytodetritus showed a more puzzling pattern. The highest inorganic carbon fixation rates were found in surface microcosms C2 and D and were correlated to exoenzymatic activities [Spearman’s Rho: BGLU = 0.50, LIP = 0.32, AMA = 0.52; *p* (fdr) <0.05; *n* = 32]. Similar findings are reported in Baltar et al. (2016), where DDIC fixation rates and associated transcripts were enhanced following a sudden intensification of bacterial heterotrophic metabolism. Indeed, DDIC is not a prerogative pathway of autotrophs (photo- or chemo-) but heterotrophs can take up and effectively use CO_2_ through metabolic pathways implicated in the synthesis of fatty acids, nucleotides, and amino acids, as well as in anaplerotic reactions (Alonso-Sáez et al., 2010), making the heterotrophic CO_2_ assimilation a relevant process for the global carbon cycle (Erb, 2011). Therefore, we hypothesize that the observed steep increase in DDIC fixation rates in microcosms C2 and D was mainly due to an intensified anaplerotic activity, deputed to fuel the intense heterotrophic activity. Moreover, representatives of *Polaribacter*, *Colwellia* and *Pseudoalteromonas* genera have been shown to significantly contribute to DIC uptake (DeLorenzo et al., 2012). These taxa were preponderant members of the community in the enclosures with higher rates of dark DIC fixation, further corroborating our hypothesis of the prevalence of anaplerotic DDIC fixation. It is also worth mentioning that the putatively chemosynthetic taxa *Nitrosopumilus* (Thaumarchaeota), *Nitrospina* and LS-NOB (Nitrospinaceae) decreased in relative abundance along with time and detritus concentration, thus suggesting a tuning of DIC uptake from (at least partially) autotrophic to heterotrophic pathways in our 4-day incubations.

Remarkably, we found significant positive correlations between all the metabolic activities tested and attached prokaryotes [Spearman’s rho: BGLU = 0.42; LIP = 0.41; AMA = 0.55; HCP = 0.29; DDIC = 0.51; *p* (fdr) <0.01; n = 96 for BGLU, LIP, AMA and HCP; *n* = 32 for DDIC] suggesting that detrital particles represented a significant hotspot of prokaryotic activity during our incubations.

## Conclusion

We hypothesized that in response to phytodetrital features and concentration, distinct microbial communities would show a different structural and functional response. The artificially generated phytodetritus well captured the peculiar duality of the phytoplankton in the Ross Sea, which is either dominated by Haptophytes (e.g., *Phaeocystis* spp.) or diatoms (Smith et al., 2014). Amendments with diatom-derived POM induced marked shifts in both surface and bottom communities, led by a consistent increase of Alteromonadales. Enrichments with *Phaeocystis-*derived material produced different effects on surface and bottom communities. Mild enrichments induced a community-level response while stronger enrichments induced the growth of specific, fast-responsive, taxa (i.e., Alteromonadales and Flavobacteriales). Bottom samples showed an exact inverse pattern highlighting that small pulses of POM are rapidly exploited while more consistent loads of OM re-shuffle the whole community. Our results show that several rare or undetected taxa in the initial community became dominant in the time course of the incubation (4 days). Furthermore, diverse organic matter sources induced site-specific changes in microbial communities, selecting for specific genera which differ in their capabilities to degrade organic matter. These experiments, in combination with the present knowledge of the metabolic strategies of those taxa, suggest that free-living communities represent functional seedbanks for the degradation of particulate organic matter of detrital origin. The emergence of bacterial groups that were also abundant in environmental and experimental phytoplankton-derived organic matter enrichments in the Southern Ocean (i.e., Landa et al., 2016, 2018) emphasize the relevance of our study in shedding light on the microbial community response of this ecosystem to organic matter pulses. Finally, our study provides novel insights on the mechanisms underlying the prokaryotic utilization of detrital particles in the mesopelagic realm, that harbor an overlooked, but significant, pool of organic matter in the dark ocean.

## Data Availability Statement

The 16S amplicon sequences generated for this study can be found in Sequence Reads Archive (SRA) at NCBI with the accession number PRJNA609227. The dataset generated for this study is available on request to the corresponding author.

## Author Contributions

MC, FM, and AV designed the experiments. VM, FC, FD, RS, AF, and MC performed the laboratory analyses. VM, EB, FD, PD, and MC processed the data. VM wrote the manuscript with the help and inputs of all co-authors.

## Conflict of Interest

The authors declare that the research was conducted in the absence of any commercial or financial relationships that could be construed as a potential conflict of interest.
