# Supplementary material for: Prokaryotic Response to Phytodetritus-Derived Organic Material in Epi- and Mesopelagic Antarctic Waters
Source: Front Microbiol. 2020 Jun 9;11:1242. doi: 10.3389/fmicb.2020.01242 (PMC7296054; doi:10.3389/fmicb.2020.01242)
Supplement: Supplementary file 1 [file Data_Sheet_1.docx]

Supplementary Material

# Supplementary Methods

Particulate Organic Carbon (POC) concentrations in plankton samples were measured using an elemental analyzer CHNO-S Costech mod. ECS 4010 applying the method by Pella and Colombo (1973). Three replicates of 3 mL were used for the analysis in each of the four plankton samples. POC was collected onto precombusted GF/F filters and stored frozen at -20 °C. Before the analyses, the filters were treated with the addition of 200 µL 1 N HCl to remove the carbonates (Lorrain *et al.*, 2003) and then dried in oven at 60 °C for about 1 h. Subsequently, sample and blank filters were folded and put on a 9x10 mm tin capsule. Known amounts of standard Acetanilide (C_8_H_9_NO – Carlo Erba; Assay ≥99.5 %) were used to calibrate the instrument.

Samples for dissolved organic carbon (DOC) concentrations in plankton samples were obtained by filtering 3 mL aliquots (in 3 replicates) through GF/F membranes and by collecting the filtrate in 20 mL glass vials, subsequently stored at -20°C. The membranes, the vials and the glassware used for the filtration had been precombusted at 480°C for 4h. Before the analyses, samples were diluted 20 times with MilliQ water, automatically acidified to pH < 2 using 6 N HCl solution (1% v/v) and sparged (150 mL min^-1^) with high-purity oxygen for 8 min.

DOC analyses were performed by the high temperature catalytic oxidation method using a Shimadzu TOC-V CSH (Cauwet, 1994). One hundred µL of sample was injected. Carbon concentration was determined by automatic comparison with four-point calibration curves. Standardization was carried out using potassium hydrogen phthalate. Each value was determined from a minimum of three injections, with a coefficient of variation < 2%.

For phytoplankton in situ analysis, samples were collected by Niskin bottles at discrete depths (surface and bottom) and fixed with pre-filtered and neutralized formaldehyde (1.6% f.c., Throndsen, 1978). Depending on phytoplankton densities, a variable volume of seawater (10–100 mL) was allowed to settle in an Utermöhl chamber and examined following the Utermöhl method (Utermöhl, 1958). Cell counts were performed using an inverted light microscope (LEICA DMi8) equipped with phase contrast along transects (1–4) at a magnification of 400× counting a minimum of 200 cells. Half or the whole Utermöhl chamber was further examined at a magnification of 200×, to obtain a more correct evaluation of less abundant microphytoplankton (>20 µm) taxa.

# Supplementary results

## Microplankton *in situ* distribution

The surface layer was characterized by high phytoplankton abundances (from 8.4 × 10^5^ cells L^-1^, at station C1, to 8.1 × 10^6^ cells L^-1^, at station D). At the bottom, abundances were not higher than 2.0 × 10^3^ cells L^-1^. In the surface layer of the station D, the phytoplankton community was dominated by diatoms (up to 92.0% of the total abundance), while dinoflagellates and flagellates accounted for 1.2 and 7.0% of the total, respectively. Among diatoms, species belonging to the genera *Pseudo-nitzschia* and *Fragilariopsis* were the most abundant (4.9 and 2.0 × 10^6^ cells L^-1^, respectively). Stations C2 and C1 were characterized by similar percentages of diatoms and flagellated forms. At the station B, a different phytoplankton community was present, being dominated by flagellated forms (92.3% of the total), among which the haptophyte *Phaeocystis antarctica* was the most abundant taxon (4.1 × 10^6^ cells L^-1^). Heterotrophic flagellates belonging to the choanoflagellate group were present in relatively high abundances, up to 4.1 × 10^5^ cells L^-1^. Diatoms and dinoflagellates accounted for 5.8 and 2.0% of the total, respectively. In the deep layers, dinoflagellates represented a high percentage (on average, 58.3% of the total abundance and up to 84.8% at the station C1), compared to the surface layer (on average, 2.7%).

# Supplementary Figures and Tables

## Supplementary Figure 1


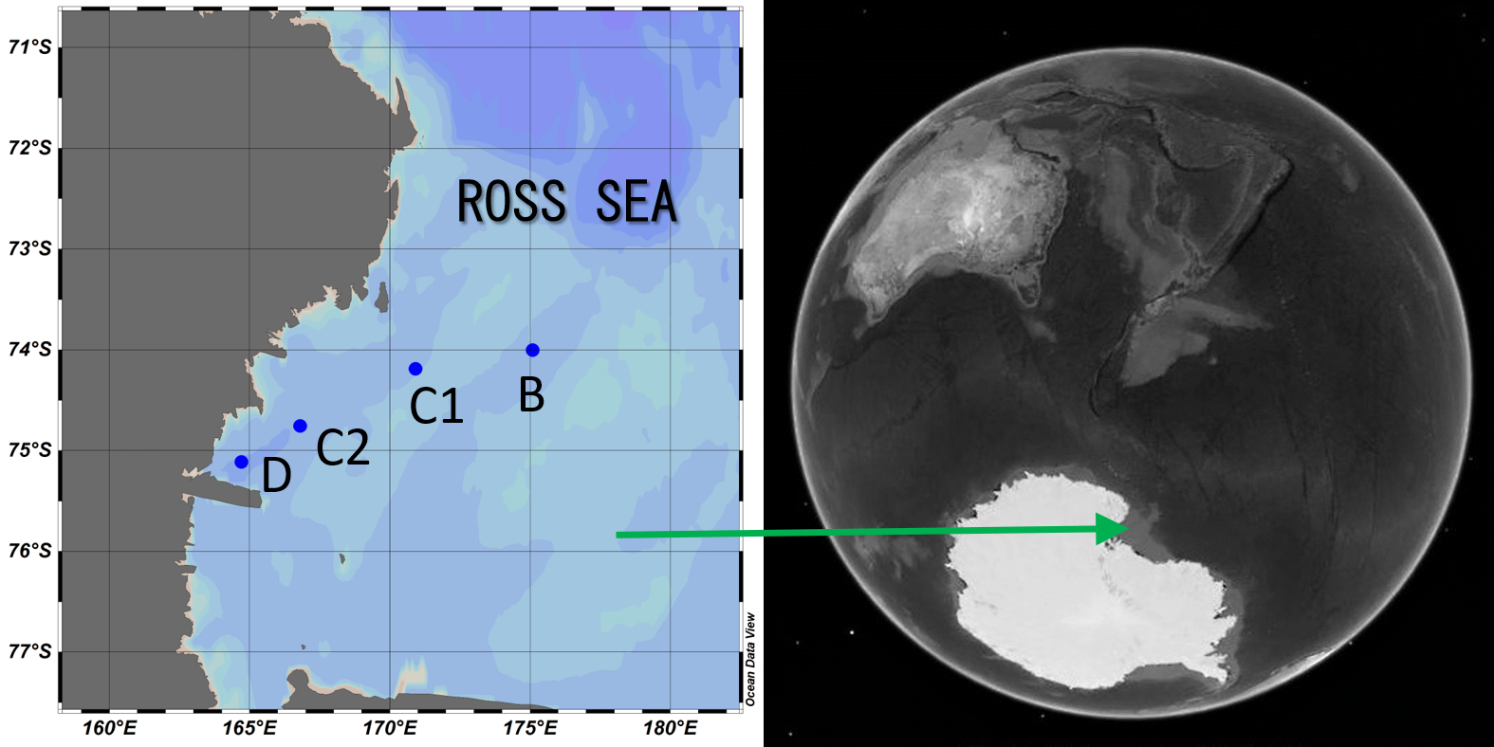


Supplementary Figure 1 Map of the sampling stations in the Ross Sea (Southern Ocean). The map was created by means of the ODV (Schlitzer, 2014) and the Google Earth software. Station bottom depths are reported in the Table 1 of the main text.

## Supplementary Figure 2


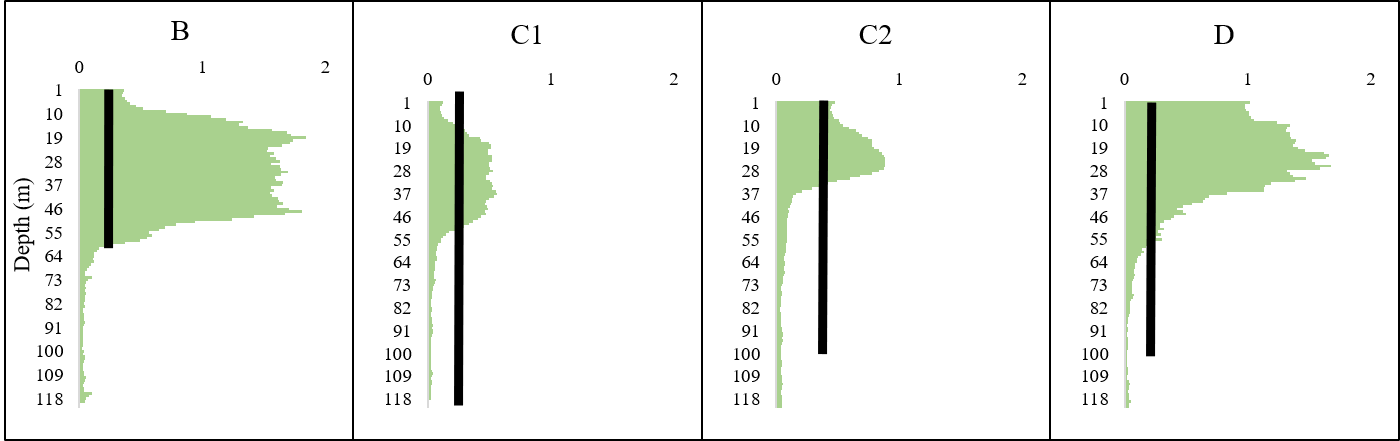


**Supplementary Figure 2.** Chlorophyll *a* fluorescence profiles (CTD) at the four stations. The black bars mark the section of the water column where the plankton net was deployed.

## Supplementary Figure 3


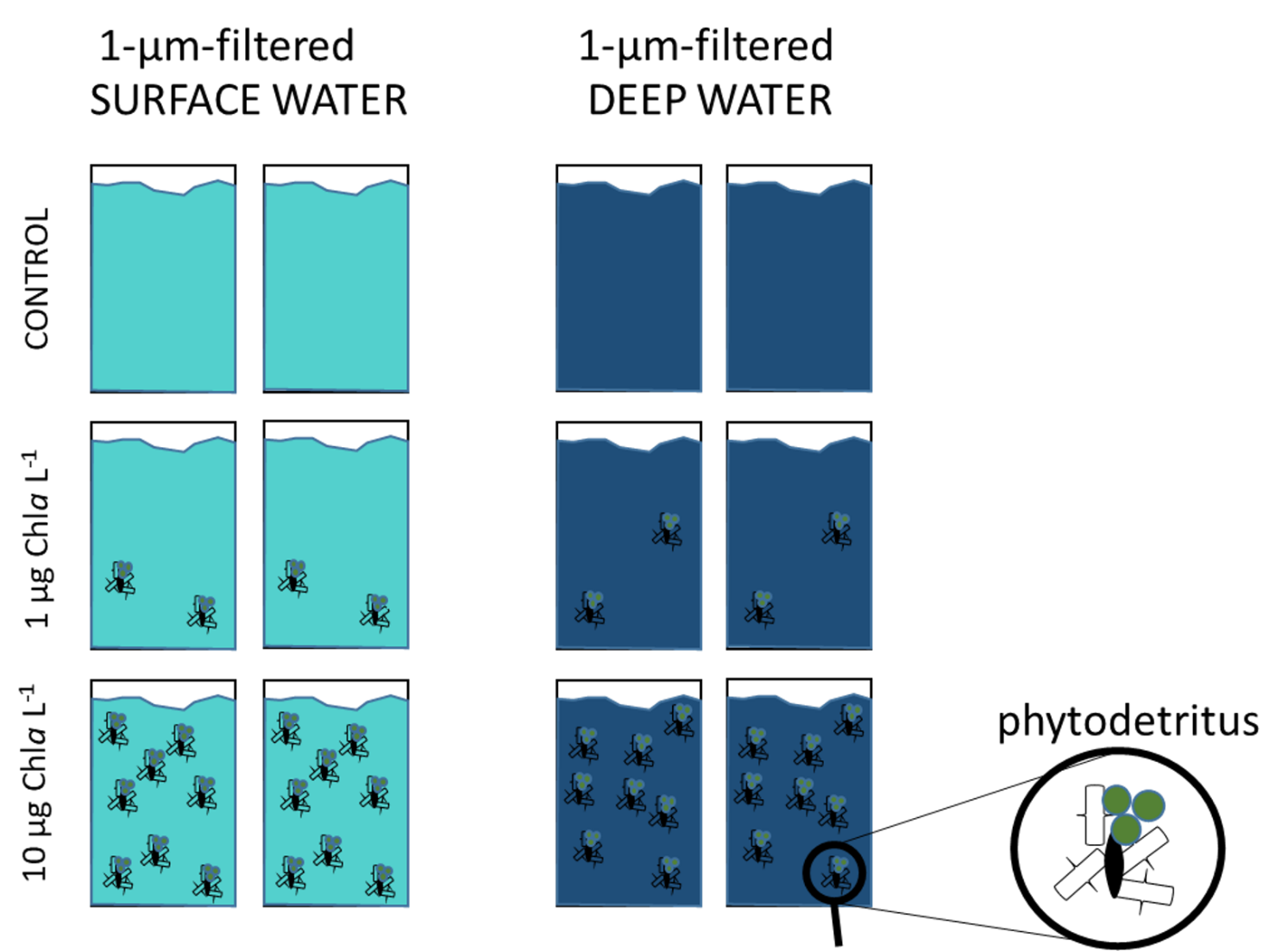


Supplementary Figure 3 Conceptual scheme depicting the experimental design of microcosms experiments at each station. Treatment and control microcosms were incubated in the dark, at *in situ* temperature for four days. The detailed experimental design is described in Section 2.2 of the main text.

## Supplementary Figure 4


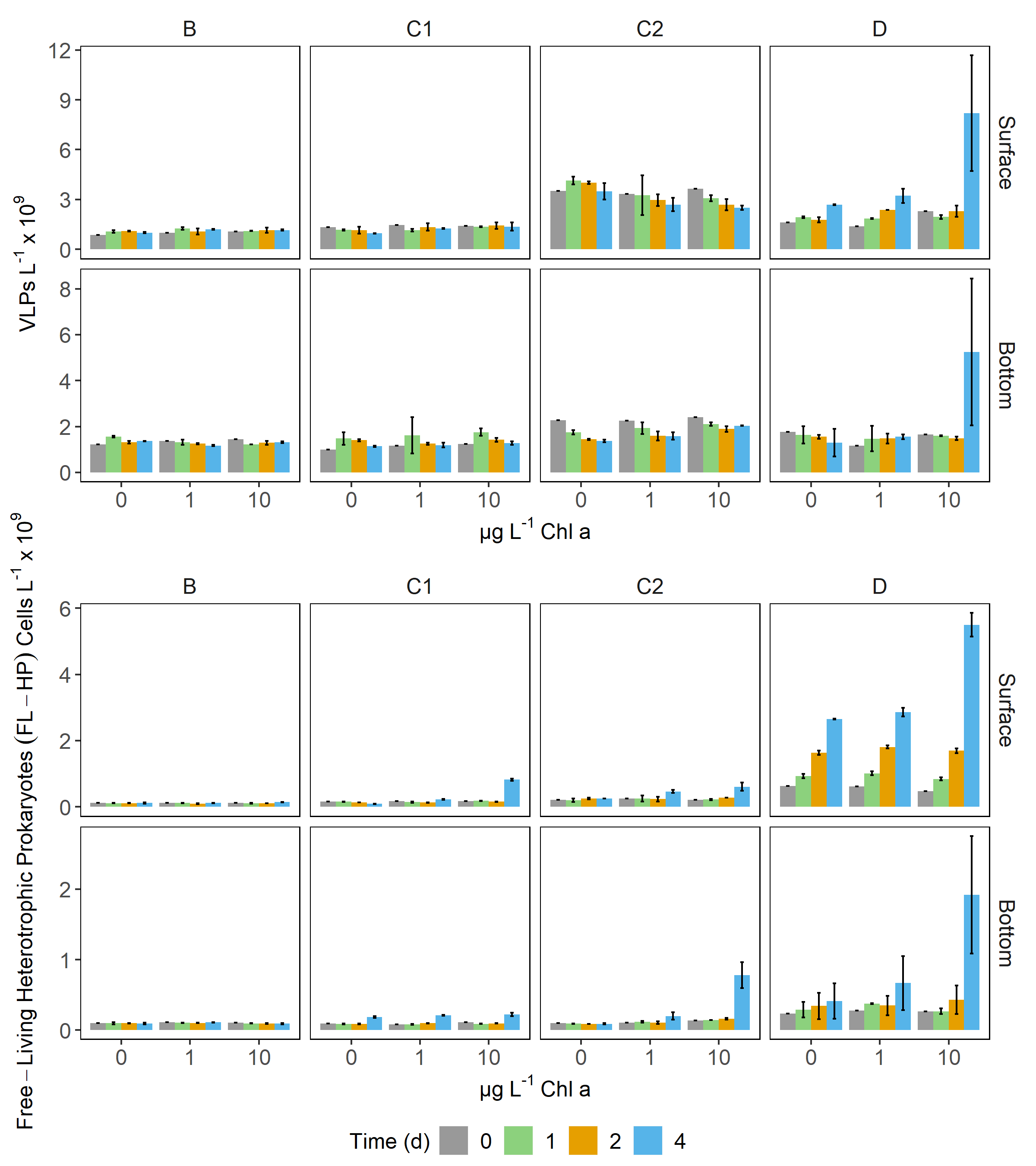


Supplementary Figure 4 Bar plots showing the abundance of virus-like particles (VLPs, upper panel) and of free-living heterotrophic prokaryotes (FL-HP, lower panel) over time in control and amended microcosms (0, 1 and 10 µg L^-1^ Chl *a*). Note that Y-axes are differentially scaled. Error bars represent the standard deviation of two experimental replicates.

## Supplementary Figure 5


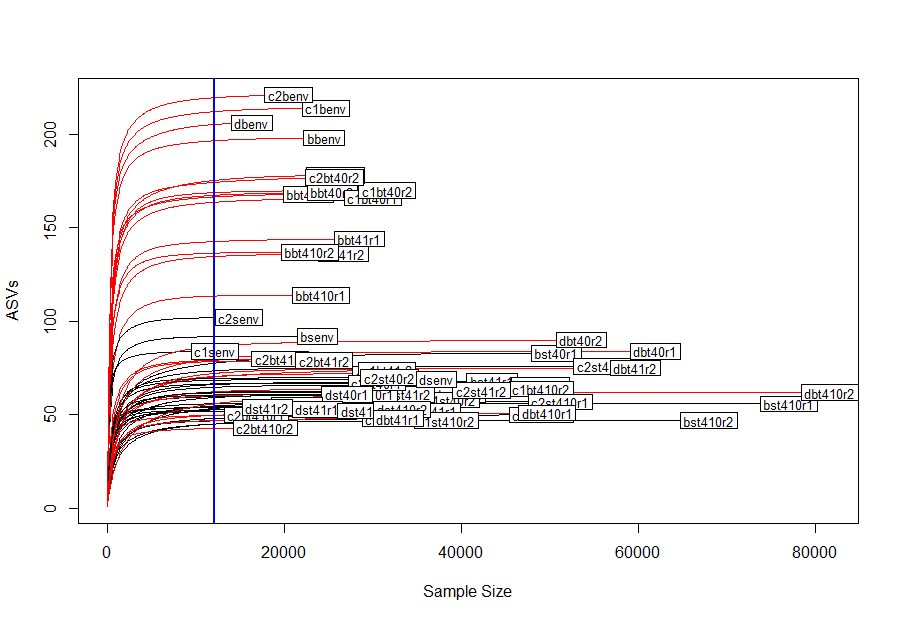


Supplementary Figure 5 Rarefaction curves of observed ASVs calculated on the non-normalized ASV table. The blue line marks the minimum number of reads (12110), retrieved in C2 surface sample on d0 (C2Senv). Surface samples are marked by black lines, bottom samples by red ones.

## Supplementary Figure 6


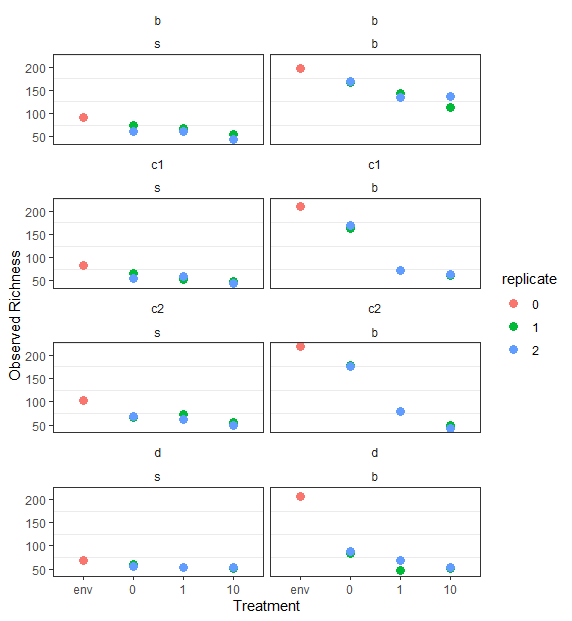


Supplementary Figure 6 Observed richness (i.e., number of unique ASVs) in the investigated samples. The x axis maps to different treatments: env= initial community, 0= control bottles, 1= bottles amended with 1 μg L^-1^, 10= bottles amended with 10 μg L^-1^. Different colors map to the different experimental replicates (0= initial (t0) community).

## Supplementary Figure 7


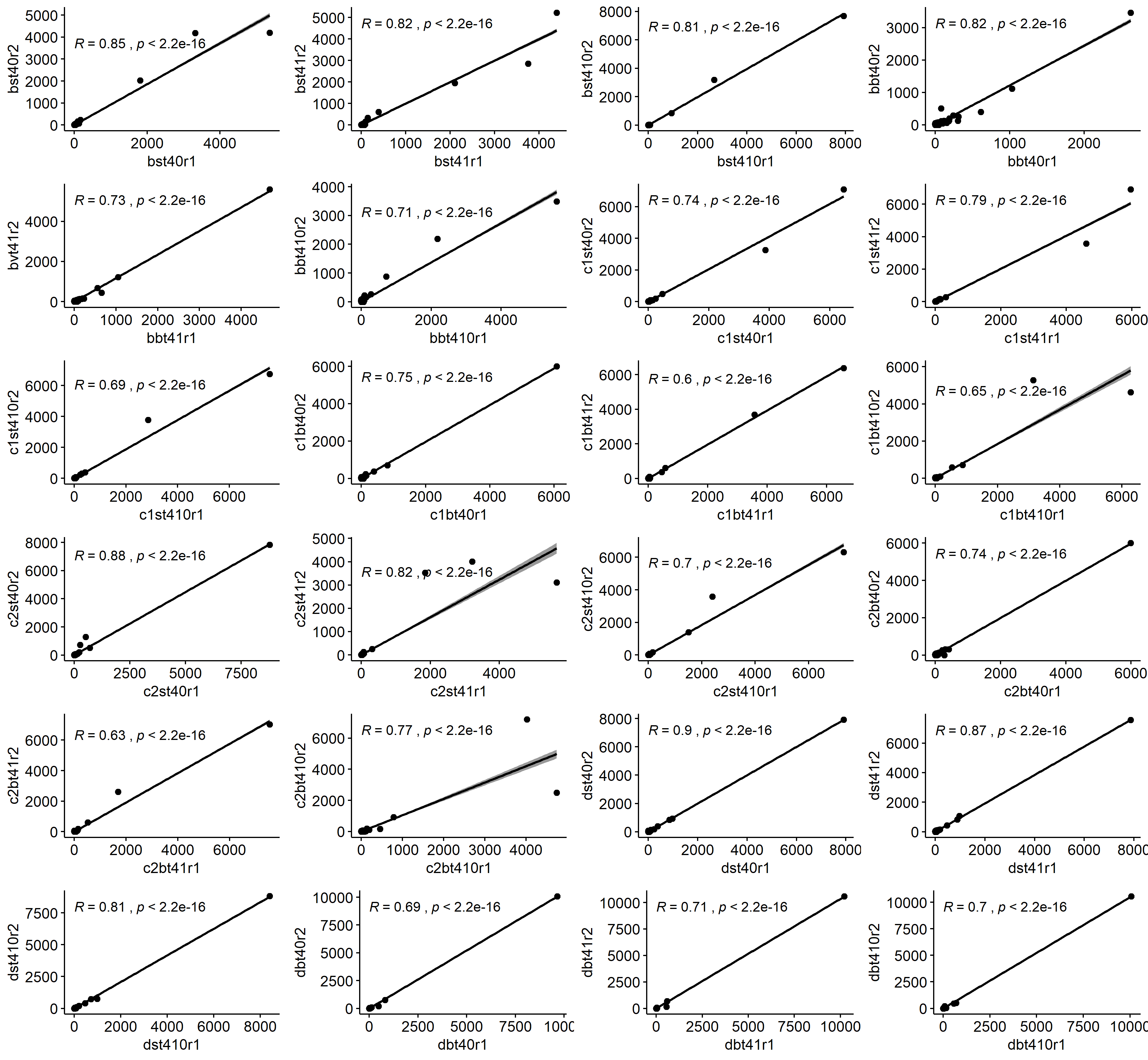


Supplementary Figure 7 Scatterplots of Spearman’s rank correlations of sequencing duplicates (raw ASVs table). Spearman’s rho and *p*-value are shown in each plot. Samples are identified according to the following code: station_depth_time_treatment_replicate. For example, replicate 1 of surface sample of station B enclosures amended with 1 µg L^-1^ Chl *a* equivalent of detritus would be identified as bst41r1.

## Supplementary Figure 8

##
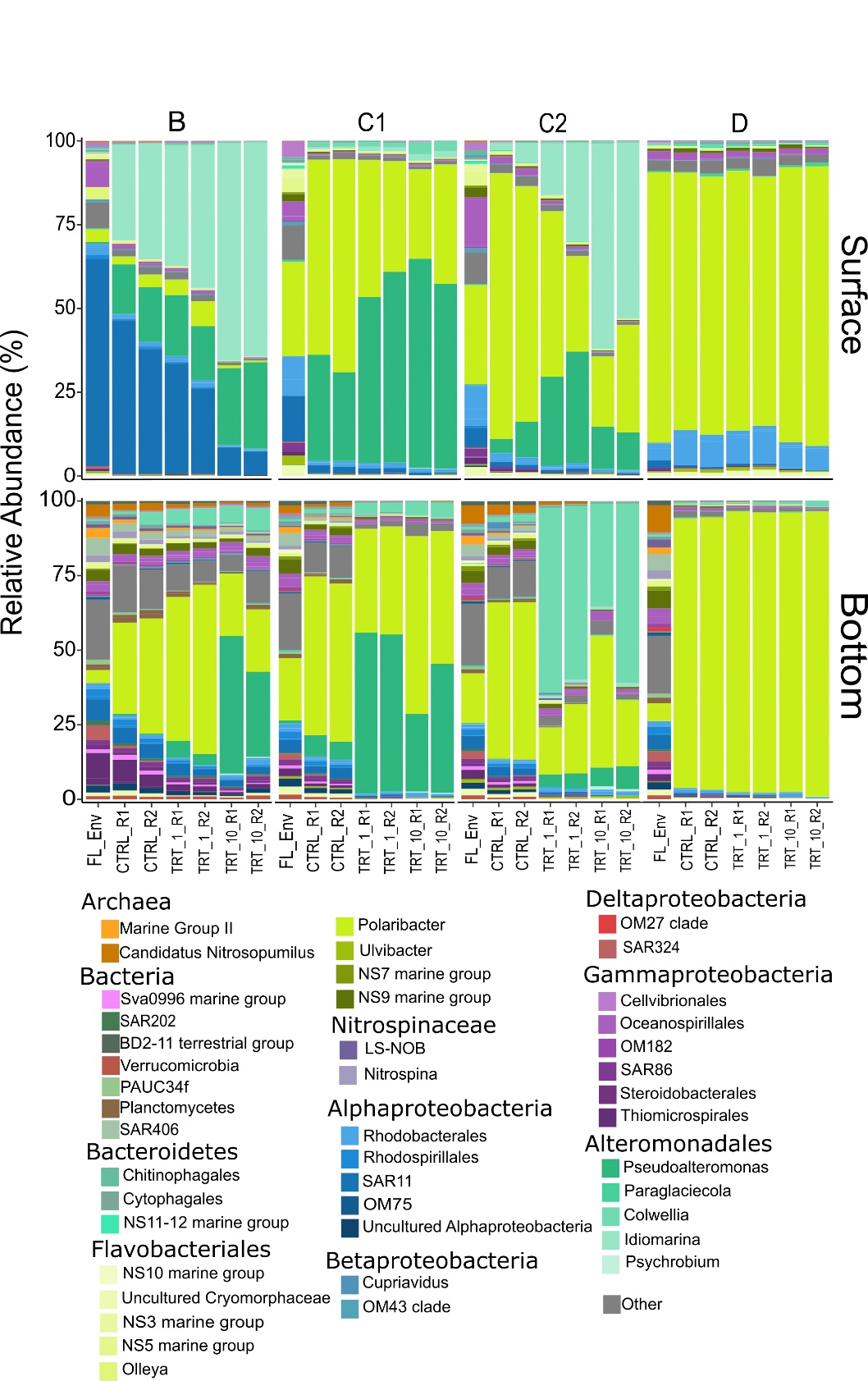


Supplementary Figure 8 Relative abundance plots of major taxa (>1% in at least one sample). FL_Env: initial free-living (1 µm-filtered) community, CTRL: control samples; TRT_1: amendments at 1 µg L^-1^ Chl *a*; TRT_10: amendments at 10 µg L^-1^ Chl *a*; R1 and R2 identify the two experimental replicates.

## Supplementary Figure 9


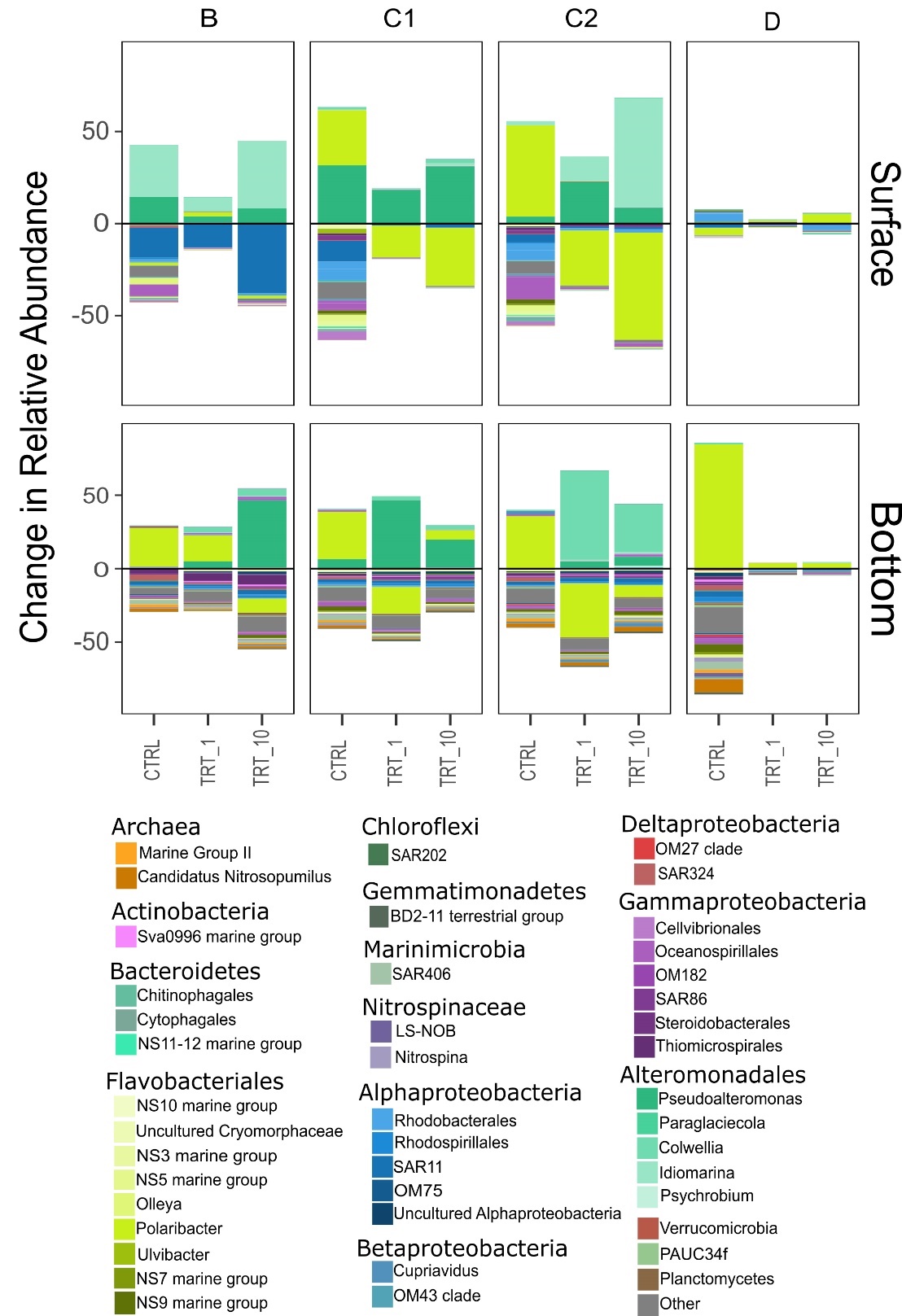


Supplementary Figure 9 Treatment-related shifts in prokaryotic community. Shifts in controls are compared to the initial (d0, 1 µm-filtered) community, whereas taxa shifts in treatments (i.e., 1 and 10 µg L^-1^) are compared against the controls (both on d4). CTRL: control samples; TRT_1: amendments at 1 µg L^-1^ Chl *a*; TRT_10: amendments at 10 µg L^-1^ Chl *a*

## Supplementary Table 1

Table 1 Results of chemical analysis on phytodetritus samples. The units of measure are reported in parenthesis. DOC and POC are presented as mean ± SD.

| **Phytodetritus** | **Chl *a* (mg L^-1^)** | **DOC (mgC L^-1^)** | **POC (mgC L^-1^)** | **POC:Chl *a*** |
| --- | --- | --- | --- | --- |
| B | 2.38 | 27.41 ± 0.68 | 52.29 ± 1.63 | 21.97 |
| C1 | 0.58 | 24.43 ± 0.48 | 49.81 ± 5.84 | 85.88 |
| C2 | 0.20 | 36.52 ± 2.51 | 41.81 ± 5.89 | 209.05 |
| D | 0.78 | 27.78 ± 0.61 | 34.47 ± 0.81 | 44.192 |

Chl *a*: Chlorophyll *a*; DOC: dissolved organic carbon; POC: particulate organic carbon.

## Supplementary Table 2

Table 2 Concentration of DOC and POC in environmental samples and added with phytodetritus amendments and calculated enrichment factors of DOC and POC in the microcosms.

| **Bottle** | **Depth** |  | **Environmental DOC*  (mgC L^-1^)** | **Added DOC (mgC L^-1^)** | | **Calculated POC ** (mgC L^-1^)** | **Added POC  (mgC L^-1^)** | | **EF DOC** | | **EF POC** | |
| --- | --- | --- | --- | --- | --- | --- | --- | --- | --- | --- | --- | --- |
|  |  | **Treatment** |  | **1** | **10** |  | **1** | **10** | **1** | **10** | **1** | **10** |
| B | Surface | | 0.76 | 0.01 | 0.12 | 0.002 | 0.02 | 0.22 | 1.0 | 1.2 | 14.9 | 139.7 |
|  | Bottom | | 0.75 | 0.01 | 0.12 | 0.001 | 0.02 | 0.22 | 1.0 | 1.2 | 18.5 | 176.1 |
| C1 | Surface | | 1.19 | 0.04 | 0.43 | 0.002 | 0.08 | 0.82 | 1.0 | 1.4 | 40.5 | 396.2 |
|  | Bottom | | 1.42 | 0.04 | 0.43 | 0.001 | 0.08 | 0.82 | 1.0 | 1.3 | 67.7 | 667.7 |
| C2 | Surface | | 0.83 | 0.09 | 0.91 | 0.003 | 0.11 | 1.13 | 1.1 | 2.1 | 40.7 | 398.2 |
|  | Bottom | | 0.69 | 0.09 | 0.91 | 0.001 | 0.11 | 1.13 | 1.1 | 2.3 | 91.5 | 906.3 |
| D | Surface | | 0.87 | 0.04 | 0.36 | 0.008 | 0.04 | 0.45 | 1.0 | 1.3 | 6.5 | 56.0 |
|  | Bottom | | 1.16 | 0.04 | 0.36 | 0.003 | 0.04 | 0.45 | 1.0 | 1.4 | 15.7 | 148.2 |

DOC: dissolved organic carbon; POC: particulate organic carbon; EF: enrichment factor, calculated as (Hardy et al., 2005).
*F. Relitti, unpublished data.
**POC concentration calculated converting prokaryotic abundance in 1µm-filtered samples assuming 13 fgC Cell^-1^ (Carlson et al., 1999). See main text for details.

## Supplementary Table 3

Table 3 Analysis of variance output of the GLM (negative binomial) models indicating the significance of phytodetrital features on the number of attached prokaryotes. As the residuals of the GLM on d1 data showed a non-normal distribution, those data were not further discussed. Significant (<0.05) P-values are highlighted in bold.

| **Variable** | **df** | **Deviance** | **Residual Df** | | **Residual Dev** | | **P-value** | **Shapiro test for residuals (p-value)** |
| --- | --- | --- | --- | --- | --- | --- | --- | --- |
| **d0** |  |  | |  | |  |  |  |
| NULL | NA | NA | | 31 | | 41.8 | NA |  |
| Pseudo-nitzschia | 1 | 5.59 | | 30 | | 36.21 | **0.02** |  |
| Phaeocystis | 1 | 0.62 | | 29 | | 35.6 | 0.43 | 0.07 |
| Chaetoceros | 1 | 1.95 | | 28 | | 33.64 | 0.16 |  |
| Choanoflagellates | 0 | 0 | | 28 | | 33.64 | NA |  |
| **d1** |  |  | |  | |  |  |  |
| NULL | NA | NA | | 31 | | 64.75 | NA |  |
| Pseudo-nitzschia | 1 | 29.83 | | 30 | | 34.92 | **<0.001** |  |
| Phaeocystis | 1 | 0.64 | | 29 | | 34.28 | 0.424 | **0.03** |
| Chaetoceros | 1 | 2.23 | | 28 | | 32.06 | 0.136 |  |
| Choanoflagellates | 0 | 0 | | 28 | | 32.06 | NA |  |
| **d4** |  |  | |  | |  |  |  |
| NULL | NA | NA | | 31 | | 61.85 | NA |  |
| Pseudo-nitzschia | 1 | 17.57 | | 30 | | 44.28 | **<0.001** |  |
| Phaeocystis | 1 | 6.91 | | 29 | | 37.38 | **0.0085** | 0.38 |
| Chaetoceros | 1 | 2.91 | | 28 | | 34.47 | **0.0088** |  |
| Choanoflagellates | 0 | 0 | | 28 | | 34.47 | NA |  |

**Supplementary References**

1. Carlson, C.A., Bates, N.R., Ducklow, H.W., Hansell, D.A. (1999). Estimation of bacterial respiration and growth efficiency in the Ross Sea, Antarctica. *Aquat. Microb. Ecol.* 19, 229–244.
2. Cauwet, G. (1994) HTCO method for dissolved organic carbon analysis in seawater: influence of catalyst on blank estimation. *Mar. Chem.* 47: 55-64.
3. Hardy, J.T., Hunter, K.A., Calmet, D., Cleary, J.J., Duce, A.R., Forbes, T.L. et al. (2005) “Report Group 2–Biological effects on chemical and radiative change in the sea surface”, in The sea surface and global change, eds. P.S. Liss and R.A. Duce (Cambridge: Cambridge University Press), 35 – 70.
4. Lorrain, A., Savoye, N., Chavaud, L., Paulet, Y.M. and Naulet, N. (2003). Decarbonation and preservation method for the analysis of organic C and N contents and stable isotope ratio of low-carbonated suspended particulate material. Anal Chim Acta 491: 125-133.
5. Pella, E. and Colombo, B. (1973) Study of carbon, hydrogen and nitrose determination by combustion-gas chromatography. *Mikrochim. Acta* 5: 697-719.
6. Schlitzer, R. (2014) Ocean data view, <http://odv.awi.de>
7. Throndsen, J. (1978) “Preservation and storage”, in Phytoplankton Manual ed. A. Surnia (Unesco, Paris), 69-74
8. Utermöhl, H., 1958. Zur Vervollkommnung der quantitative Phytoplankton-Methodik: mit 1 Tabelle und 15 abbildungen im Text und auf 1 Tafel. Internationale Vereinigung für theoretische und angewandte Limnologie: Die Mitteilungen 9, 1–38
